# Mycotoxins in Cereal-Based Products and Their Impacts on the Health of Humans, Livestock Animals and Pets

**DOI:** 10.3390/toxins15080480

**Published:** 2023-07-28

**Authors:** Jianmei Yu, Ivana Ramos Pedroso

**Affiliations:** Department of Family and Consumer Sciences, North Carolina Agricultural and Technical State University, 1601 East Market Street, Greensboro, NC 27411, USA

**Keywords:** cereal grains, mycotoxins, health impacts, human health, livestock animals, pets, management, regulation

## Abstract

Cereal grains are the most important food staples for human beings and livestock animals. They can be processed into various types of food and feed products such as bread, pasta, breakfast cereals, cake, snacks, beer, complete feed, and pet foods. However, cereal grains are vulnerable to the contamination of soil microorganisms, particularly molds. The toxigenic fungi/molds not only cause quality deterioration and grain loss, but also produce toxic secondary metabolites, mycotoxins, which can cause acute toxicity, death, and chronic diseases such as cancer, immunity suppression, growth impairment, and neural tube defects in humans, livestock animals and pets. To protect human beings and animals from these health risks, many countries have established/adopted regulations to limit exposure to mycotoxins. The purpose of this review is to update the evidence regarding the occurrence and co-occurrence of mycotoxins in cereal grains and cereal-derived food and feed products and their health impacts on human beings, livestock animals and pets. The effort for safe food and feed supplies including prevention technologies, detoxification technologies/methods and up-to-date regulation limits of frequently detected mycotoxins in cereal grains for food and feed in major cereal-producing countries are also provided. Some important areas worthy of further investigation are proposed.

## 1. Introduction

Mycotoxins are small organic molecules synthesized as secondary metabolites by certain fungal species that may contaminate various agriculture commodities such as cereals, corn, nuts, spices, soybeans and coffee beans, among others. Aflatoxins (AFB_1_, B_2_, G_1_ and G_2_), ochratoxin A (OTA), citrinin, patulin, trichothecenes (mainly deoxynivalenol (DON), T2-toxin (T_2_) and HT2-toxin (HT_2_)), fumonisins (FB_1_, FB_2_ and FB_3_) and zearalenone (ZEA) are the most prevalent mycotoxins in cereal crops [1]. Mycotoxin contamination of cereal grains remains the main food safety issue. These toxins are produced primarily by fungi genera *Aspergillus*, *Fusarium*, *Penicillium*, and *Alternaria* [2]. Aflatoxins are generated by *A. flavus and A. parasiticus*, while ochratoxin A can be produced by *Aspergillus* spp. [3] and *Penicillium* spp. [4]. Fumonisins, trichothecenes and ZEA are produced by *Fusarium* spp. and are collectively called fusarium mycotoxins. One mycotoxin can be produced by several fungi species and some mold species can produce multiple mycotoxins; nevertheless, toxic fungal development is not always accompanied by mycotoxin production [5]. The fusarium mycotoxins may cause significant pre-harvest grain loss, while aflatoxins and OTA are mostly occurring during storage due to improper postharvest handling and storage [6,7].

Mycotoxins are hazardous to both human and animal health [8]. They can cause acute toxicity and chronic diseases depending on the dosages. Acute intoxication occurs when highly contaminated food/feed is ingested in large quantities, which often results in death, as happened in Western India in 1974, Kenya from 1981 to 2006, Tanzania in 2016, Taiwan and Malaysia [9,10,11,12,13]. Long-term exposure to mycotoxins at doses slightly above the regulation limits can lead to chronic diseases such as liver cancer caused by AFs in Africa and China [14,15,16,17,18,19], kidney diseases caused by OTA in Europe and Egypt, and esophageal cancer caused by FUMs in Africa and some regions of China [20,21,22,23]. There has been a wide variety of toxic effects in both animals and humans from oral intake of mycotoxin-contaminated foods/feeds, such as immunosuppression, genotoxic, teratogenic or cancerous mutagenesis.

Among the mycotoxins that commonly occurred in cereal grains, aflatoxins, including AFB_1_, AFB_2_, AFG_1_, AFG_2_ and AFM_1_, are considered the most toxic and have been proven to be human carcinogens; thereby, they are classified as group 1 carcinogens, while FB_1_, FB_2_ and OTA are carcinogenic in tested animals, but there is not sufficient evidence about their carcinogenicity in human, and thus they are classified as group 2B carcinogens [24]. Despite health impacts, it has been shown that these contaminants can cost billions of dollars every year. In underdeveloped countries, aflatoxins have impacted around 4.5 billion individuals and aflatoxicosis is ranked sixth among the top 10 health threats [25]. Zearalenone (ZEA) has been reported to have immunotoxic, hepatotoxic and xenogenic effects [26]. Trichothecenes are divided into four groups: types A, B, C and D, with type A and B trichothecenes being the most prominent toxins in barley, wheat, oats and maize. Studies have shown that type A trichothecenes are more toxic than type B, while type B trichothecenes are present in contaminated cereals at a higher concentration than trichothecenes A [4,14]. The A group includes but is not limited to T-2 and HT-2 toxins, while the B group mainly includes deoxynivalenol (DON), nivalenol (NIV), 3-acetyldeoxynivalenol (3Ac-DON), 15-acetyldeoxynivalenol (15Ac-DON) and fusarenone X [4,27].

The cereal grains can be contaminated by these mycotoxins in the field (before harvest) or during post-harvest handling and storage. Some cereals often contain more than one mycotoxin. To protect consumers from mycotoxicoses, many countries have established or adopted regulations to limit exposure to mycotoxins. However, the adopted standards among different nations or multilateral agencies vary widely due largely to the level of economic development and the susceptibility of a nation’s crops to contamination [28]. Studies indicate that the economic costs of standard enforcement and the loss of trade opportunities resulting from the differences in allowable levels of mycotoxins in grains and grain-derived products are substantial. In developing countries, improving food safety along Western standards may result in considerable costs, thus unaffordable high food prices for low-income populations [29].

This review provides some of the more recent work on the occurrence and co-occurrence of major mycotoxins in cereal grains, as well as grain-based food/feed, the human and animal health risks caused by these mycotoxins and the management strategies to ensure the safety of cereal-based food and feed. Future research needed to identify and better understand the health problems caused by co-exposure to multiple mycotoxins and long-term exposure to lower-dose mycotoxins are also considered in this article.

## 2. Common Mycotoxins in Cereal Grains and Their Producing Fungi

### 2.1. Aflatoxins and Their Producing Fungi

**Figure 1:**
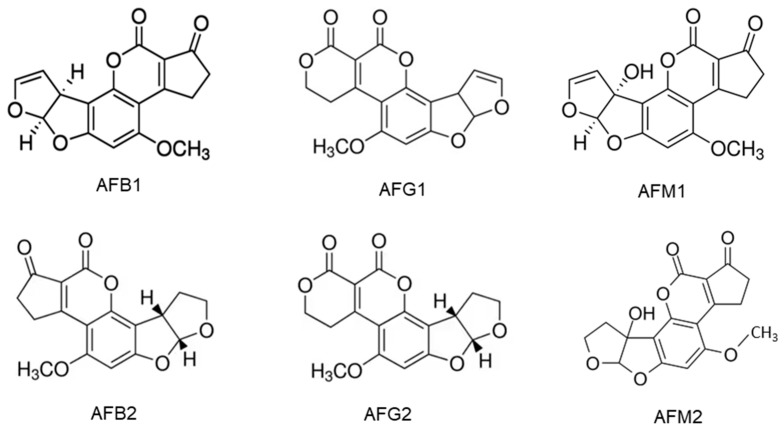
Chemical structures of aflatoxins B_1_, B_2_, G_1_, G_2_, M_1_ and M_2_.

Aflatoxins are synthesized by *Aspergillus* flavus and *A. parasiticus. A. flavus* produces B aflatoxins, while *A. parasiticus* produces both B and G forms [3]. Aflatoxins B_1_, B_2_, G_1_ and G_2_ are naturally biosynthesized and frequently detected in cereal grains, particularly, maize, while the hydroxylated metabolites of AFB_1_ and AFB_2_ are aflatoxin M_1_ (AFM_1_) and M_2_ (AFM_2_) which are present in the meat, eggs, milk and cheese produced from animals which ingested aflatoxin-contaminated feed [30]. AFM_1_ is the major metabolite of AFB_1_ in milk from nursing humans and animals that consume AFB_1_-contaminated food or feed [4]. The chemical structures of AFs are shown in Figure 1. They are slightly soluble in water with a solubility of 10–20 µg/mL and completely soluble in moderately polar solvents such as chloroform, menthol and dimethyl sulfoxide [24]. The low hydrophilicity and high hydrophobicity of AFs enable them to bind to cell membrane lipids easily.

Aflatoxins (AFs) are cytotoxic and genotoxic. AFB_1_, AFG_1_ and AFM_1_ are carcinogenic when ingested orally via the diet or delivered by gavage. The evidence for the carcinogenicity of AFB_2_ and AFG_2_ is insufficient. The AFB_1_ is more toxic than AFG_1_ in liver carcinogenicity but AFG_1_ induced a higher incidence of kidney tumors than AFB_1_. The AFB_1_ is about 10 times more potent than AFM_1_ in causing liver carcinogenicity [31]. The AFB_1_ is genotoxic and participates in the extrahepatic cycle, leading to chromosomal abnormalities, micronucleus formation, sister chromatid exchange, unscheduled DNA synthesis and DNA strand breakage [32]. The damage to DNA ultimately leads to the development of cancer. A human cell line study shows that AFB_1_ and AFG_1_, their precursors, as well as their metabolites aflatoxicol (AFL) and AFM_1_ are genotoxic [33]. Studies with poultry have found that the AFB_1_ and its metabolites mainly target the liver, where the toxin is metabolized mainly by CYP1A2 and CYP3A4 and causes numerous mutations, particularly in the p53 tumor suppressor gene [32]. This should be at least partially responsible for the high liver cancer incidence in some regions of the world where people are frequently exposed to food products contaminated with AFs. 

### 2.2. Ochratoxins and Their Producing Fungi

**Figure 2:**
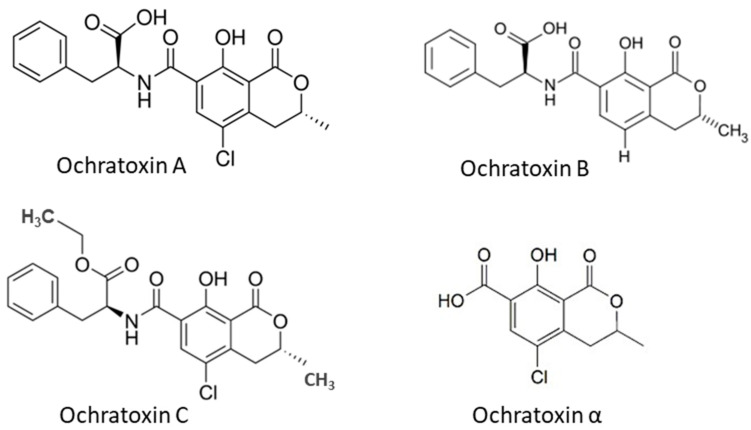
Chemical structure of ochratoxins occurred in cereal grains.

Ochratoxins (OTs) are produced by certain *Aspergillus* species and some *Penicillium* species, including *A. ochraceus*, *A. alliaceus*, *A. auricomus*, *A. carbonarius*, *A. glaucus*, *A. melleus*, *A. niger*, *P. nordicum* and *P. verrucosum* among them [4,34]. The main forms of OTs are ochratoxin A, B and C, which differ in chemical structure and toxicity. Ochratoxin B (OTB) is a non-chlorinated form of OTA and ochratoxin C (OTC) is an ethyl ester of OTA formed in the presence of rumen fluid [8]. Among these ochratoxins, OTA is the most prevalent and toxic followed by OTC and OTB [35]. OTα is a non-toxic metabolite of OTA. The OTA has been found in several types of cereals, including corn, wheat, barley, rye, rice and other plant products such as coffee beans, dried fruits and spices. The chemical structures of OTs (Figure 2) show that all OTs contain a non-polar end and several polar groups on the side chain; thus, they can bind to membrane lipids and proteins such as plasma albumin [22,36]. 

OTA is at least ten times more toxic than OTB and OTC. The OTA is nephrotoxic to all animal species and humans, and it has been related to the Balkan endemic nephropathy [37,38]. In addition, the OTA is also known to be hepatotoxic, immunotoxic, neurotoxic, teratogenic and carcinogenic, involving multiple mechanisms [35,39]. A single high dose or multiple lower doses of OTA in rats inhibits protein synthesis, mitochondrial respiration and ATP formation. It also enhances lipid peroxidation and the formation of reactive oxygen species (ROS) and reactive nitrogen species (RNS) [39,40]. High levels of ROS result in decreased activity of cellular antioxidant enzymes, thus leading to oxidative stress and further inflammatory diseases [40]. Elevated levels of RNS induce nitrosative stress associated with DNA damage, tissue toxicity, cancer and inflammatory conditions, which may be responsible for cell injury and death [41].

### 2.3. Zearalenone and Its Producing Fungi

**Figure 3:**
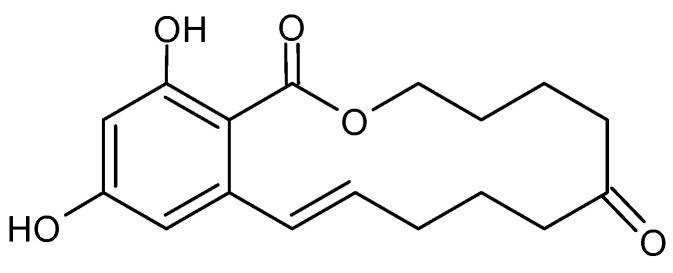
Chemical structure of Zearalenone.

ZEA is produced by fungi of the genus *Fusarium* spp., including *F. graminearum*, *F. culmorum*, *F. cerealis*, *F. equiseti*, *F. crookwellense*, *F. semitectum*, *F. verticillioides*, *F. sporotrichioides*, *F. oxysporum* and *F. acuminatum* [26], but mainly *F. graminearum* and *F. culmorum* [42]. Corn has been described as the most vulnerable food to ZEA contamination. Other food crops, including wheat, barley, oat and rye, have also been found with this toxin. ZEA is immunotoxic, hepatotoxic and xenogenic [26]. The chemical structure of ZEA consists of a resorcinol moiety fused to a 14-member macrocyclic lactone ring with a trans double bond, two hydroxyl groups, two ketones and one methyl branch (Figure 3), which allow it to be easily absorbed through the gastrointestinal tract, to interact with proteins and lipids in the biological system to exert its toxicity [43,44]. The ZEA induces histopathological changes in the liver, with the subsequent development of liver cancer; it exerts hematotoxic effects by disturbing blood coagulation and modifying blood parameters. ZEA and its derivatives are non-steroids but have estrogenic activity in mammalian animals. They bind to estrogen receptors of cells and inhibit the secretion of steroid hormones, interfere with the estrogen response in the pre-ovulatory phase, and inhibit follicle maturation in mammals; this leads to disorders of the hormonal balance of the body, and, subsequently, many diseases of the reproductive system [43,44]. Higher concentrations of ZEA cause permanent estrus, pseudo-pregnancy and infertility in animals [44]. The detail toxicity of ZEA in humans and animals has been extensively reviewed by Ropejko and Twarużek [26] and is not discussed in this review.

### 2.4. Fumonisins and Their Producing Fungi

**Figure 4:**
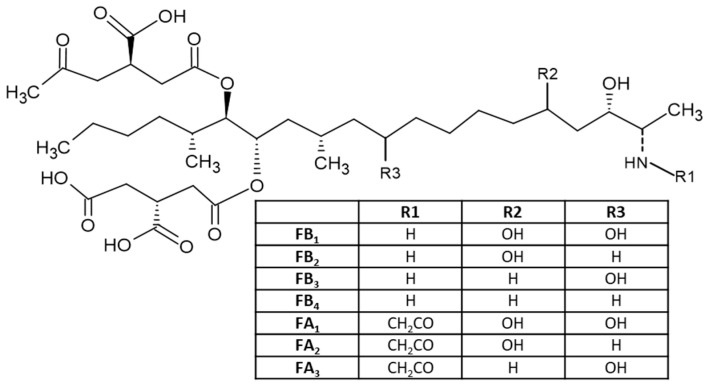
Chemical structure of different fumonisins.

Fumonisins (FUMs) are produced in cereals by different species of *Fusarium* including *F. verticillioides*, *F. proliferatum* and *F. fujikuroi*, and other related species; they are common contaminants of maize and to a lesser extent of wheat and other cereals [45]. Fumonisins consist of a long hydrocarbon backbone chain similar to that of sphinganine. Six forms of fumonisin including FA_1_, FA_2_, FB_1_, FB_2_, FB_3_ and FB_4_ have been identified, with FB_1_ being the most toxic [4]. All FUMs are water soluble and thus are polar [24], which is determined by their chemical structures (Figure 4). These toxins are cytotoxic and carcinogenic to animals at relatively high concentrations. FB_1_, the major and most-studied fumonisin, is nephrotoxic and hepatotoxic in several animal species and has been classified as a possible carcinogen to humans (Group 2B) [24]. FB_1_ has been reported to cause leukoencephalomalacia (LEM) in horses, pulmonary edema syndrome (PES) in pigs and liver cancer in rats and hepatotoxic to horses, pigs, rats and vervet monkeys. The FB_1_ is cytotoxic to mammalian cell cultures and phytotoxic to several plants. FUMs have also been reported to be linked to human esophageal cancer in Transkei, China and South Africa [20,21,22,23,46]. 

### 2.5. Trichothecenes and Their Producing Fungi

**Figure 5:**
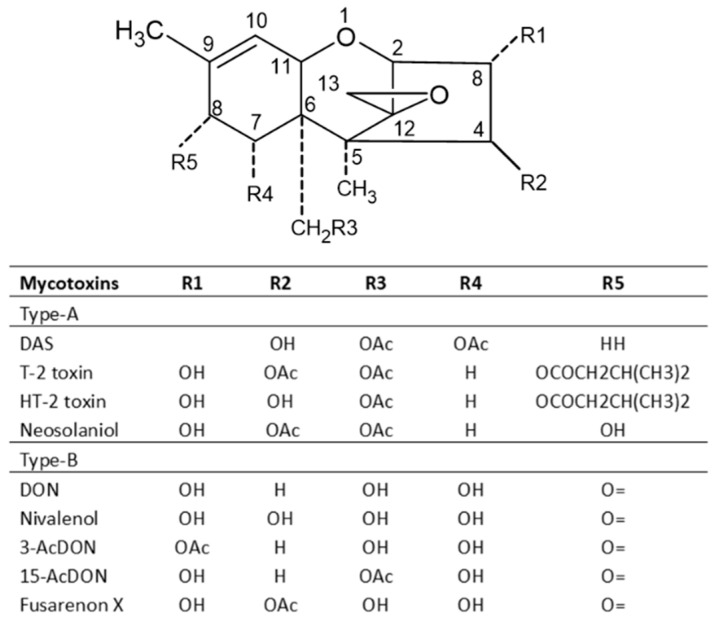
Chemical structures of major trichothecene mycotoxins.

Trichothecenes are several groups of mycotoxins produced by fungi of the *Fusarium* genus [14]. They are divided into four groups: types A, B, C and D. The type A group mainly consists of T-2 and HT-2 toxins, diacetoxy- and monoacetoxy-scirpenol (DAS and MAS) and neosolaniol (NEO). The B group mainly includes DON, nivalenol (NIV), fusarenone X and DON derivatives 3Ac-DON and 15Ac-DON [4,27]. Type C trichothecenes contain a C-7/C-8 epoxide (e.g., crotocin), while type D trichothecenes have an additional ring linking the C-4 and C-15 position (e.g., roridin A, verrucarin A and satratoxin H) [47]. Type A and B trichothecenes are the most common in barley, wheat, oats and maize. Studies have shown that the toxicity of type A trichothecenes is greater than that of type B but, fortunately, the concentrations of type A trichothecenes present in contaminated cereals are much lower than that of trichothecenes B [4]. Among trichothecene mycotoxins, the T-2 toxin is the most toxic and it is considered an immunosuppressive, cytotoxic, lymphocytic and carcinogenic mycotoxin in mammalian cells. The toxicity of the T-2 toxin is extensively reviewed by Janik et al. (2021) [48]. The chemical structures of trichothecenes are depicted in Figure 5 [49], which indicates that DON and NIV are more polar, while the T-2 toxin is hydrophobic, and the polarity of HT-2 toxin is between DON/NIV and T-2 toxin. This may contribute to the higher toxicity of T-2 toxin. However, T-2 toxin levels in food and feed are not regulated in many countries including the US.

Type A trichothecenes are manly produced by strains of *F. sporotrichioides* and *F. poae*, while type B trichothecenes toxins are mainly produced by strains of *F. culmorum* and *F. graminearum* [50]. Milder climatic conditions without high humidity favor the production of type A trichothecenes [51]. In the European maize growing areas, this toxin is usually detected in corn red ear rot. Oral exposure to T-2 toxin can lead to a fatal condition, known as alimentary toxic aleukia (ATA) with radiation poisoning-like symptoms [52]. DON and other Type B trichothecenes are not as potent in mammalian systems compared with T-2 toxin, but can still be lethal at high enough concentrations. DON is also known as ‘vomitoxin’ for its ability to cause diarrhea and emesis. DON and its derivatives cause a variety of maladies, including anorexia, feed refusal in livestock, growth retardation, leukocytosis, hemorrhage and adverse effects on reproduction and development. The toxicity of DON and its derivatives to animals is in the order of 15Ac-DON > 3Ac-DON > DON > DON-3Glc [2,53].

## 3. Occurrence and Co-Occurrence of Mycotoxins in Unprocessed Cereal Grains

### 3.1. Occurrence of Mycotoxins in Unprocessed Cereal Grains

Mycotoxin contamination can occur in the field and during storage. The hot and humid tropical and subtropical climate conditions are considered the dominant factors concerning aflatoxins, especially in developing countries where food product may not be sufficient and storage condition is poor [54]. AFs, FUMs, DON and OTA are the most commonly identified mycotoxins, especially in corn grains [55]. The number of mycotoxins in contaminated foods and feeds has been the subject of investigations in the past few decades.

Prior to 1985, the United Nations Food and Agriculture Organization (FAO) estimated that around 25% of the world’s foods contain some form of mycotoxins [56]. However, data from around 500,000 analyses from the European Food Safety Authority and data released from recent large surveys on mycotoxin occurrence across the world by Biomin suggest higher than 25% of mycotoxin prevalence [57]. For example, Streit et al. reported that, overall, 72% of 17,300 feed samples originating from different parts of the world collected over eight years contained mycotoxins [55]. An extensive literature review has found that the global mycotoxin prevalence in food crops is up to 60–80%, although the data varies largely depending on many factors, such as the mycotoxin of concern, used analytical methods and reporting of the results [57]. According to the 2020 BIOMIN World Mycotoxin Survey Report, the most prevalent mycotoxins globally are the *Fusarium* mycotoxins DON (65%) and FUM (64%), followed by ZEA (48%) in the cereal crops harvest in 2020 as shown in Table 1 [58].

### 3.2. Co-Occurrence of Mycotoxins in Unprocessed Cereal Grains

With the advancement of analytical instruments and techniques, researchers have reported an increased number of mycotoxins in various food products, with peanuts, maize and tree nuts being considered the most vulnerable crops. Rice, cottonseed, tree nuts, spices and figs are also ranked high for mycotoxin contamination [59]. The issues of co-occurrence and high concurrent exposure to AFs and FUMs in maize-eating populations in Africa, Latin America and parts of Asia have been reported [60]. The FUMs and aflatoxins are most frequently detected in maize and to a lesser extent, in rice, sorghum, wheat and cereal-based foods prepared from these cereals [45]. The degree of co-occurrence of AFs and FUMs varies with many factors, including the type of commodity, region, time of sampling, storage condition, food preparation and processing. A recent evaluation conducted by the Joint FAO/WHO Expert Committee on Food Additives [61] found co-occurrence of AFs and FUMs in 1.7% of around 5000 samples submitted to the GEMS/Food Contaminants database between 2011 and 2016. For individual samples, the co-occurrence rate was 5.5% in maize samples, 4.2% in cereals and cereal-based products, 2.8% in bread and other cooked cereal products, 1.4% in sorghum and 0.4% in cereal-based foods for infants and young children, respectively [62]. More studies regarding the co-occurrence of mycotoxins in unprocessed cereal grains and the percentage of tested samples with mycotoxin content above EU regulation limits are summarized in Table 2. 

Table 2 clearly shows that (1) most of the unprocessed cereal grains are contaminated by multiple mycotoxins no matter whether the grains are produced in developing countries or developed countries and the percentage of samples containing multiple mycotoxins is way higher than that reported in [62]. (2) Grain samples produced in developing countries often have higher AFB_1_ and OTA contents and higher percentages of samples with mycotoxin levels above the regulation limits. Certain portions of these mycotoxins will be carried into cooked/processed food and feed products if the highly contaminated grains are included in the ingredients because common cooking and processing methods (such as sorting, trimming, cleaning, milling, baking, extrusion and roasting) cannot eliminate them, although they can significantly reduce their contents [84].

## 4. Occurrence and Co-Occurrence of Mycotoxins in Cereal-Based Products

### 4.1. Mycotoxin Contamination of Cereal-Based Foods for Human Consumption 

Humans can be exposed to mycotoxins through foods and their working or living environments, but the ingestion of contaminated foods, particularly cereal-based foods and nuts, is the main cause of human mycotoxicoses [85]. The types and levels of mycotoxins in human diets also vary greatly with geographical locations and availability of specific cereal, the quantity of cereal-based food in the diet, spices and other ingredients in the diet, as well as cooking or processing methods. For example, the main cereal in the human diet is corn in Africa and South America, rice in Asia and wheat in North America and Europe, while the dominant mycotoxins in those cereals are AFs, OTA and DON, respectively, although multiple mycotoxins co-exist in one cereal, as shown in Table 2. As mentioned in Section 3.2, each step of food/feed processing and cooking can reduce certain levels of mycotoxins and result in relatively safe ready-to-eat products [84]. That is why there are very few real cases of mycotoxin poisoning. However, food processing cannot eliminate them; thus, processed/cooked foods often contain different levels of mycotoxins, mostly lower than the regulation limits, depending on the processing methods and initial mycotoxin loads. Table 3 summarizes recent findings on mycotoxins in processed foods. Overall, the prevalence and concentration of mycotoxins in finished food products in developing countries are much higher than those in developed countries. Thus, people living in developing countries such as those in Asia and Africa are at greater risk of being exposed to high levels of mycotoxins than those living in the developed countries. The low mycotoxin levels in processed foods in developed countries are due to the relatively low mycotoxin contents in unprocessed cereal grains, better storage condition control, and the implementation of regulations. This also explains the fact that human mycotoxicosis is rare in Western countries, but relatively more frequent in developing countries. 

#### 4.1.1. Aflatoxins in Cereal-Based Foods

The dietary exposure to aflatoxins in industrialized and underdeveloped countries is different. In developed countries, mean aflatoxin dietary exposures are generally lower than 1 ng/kg body weight (bw) per day, whereas the estimated dietary exposure of aflatoxin in some sub-Saharan African countries is higher than 100 ng/kg bw per day [45]. Table 3 shows that the occurrence of AFs or AFB_1_ in cereal-based food products for European countries is rare and the levels of these AFs are extremely low. Thus, human mycotoxin poisoning in a developed country is rare. This is attributed to low AFs levels in unprocessed grains, as shown in Table 2, and tighter enforcement and implementation of mycotoxin regulations in developed countries [107]. However, the occurrence and levels of AFs in cereal-based food products in the markets of developing countries are high. Table 3 also shows that corn-based food products consumed in African and some Asian countries have remarkably higher incidences and levels of AFs compared to those in other regions of the world. This may be associated with higher liver cancer incidence in developing countries, particularly in Asia and Africa.

#### 4.1.2. Ochratoxin A in Cereal-Based Foods

Human beings are exposed to OTA mainly through the consumption of various foods including cereals and cereal-based food products, fruits, juice, beer, wine and coffee, and to a lesser extent, animal products such as meats, eggs and milk products [108]. The OTA concentrations in processed cereal products in the US, Canada and Europe are usually low and do not pose health risks with the exception of infant foods, as shown in Table 3. However, higher OTA concentrations have been reported in cereal-based adult food products in some developing countries.

One study assessed the OTA content in 144 breakfast cereal and snack samples collected from six areas in the US and found that 75 samples (52%) were OTA positive with a concentration of 0.10–7.43 ng/g. Among the OTA-positive samples, 40% were organic and 60% were conventional, respectively [109]. Another survey conducted by the same research group analyzed OTA contents in 489 corn-, rice-, wheat- and oat-based breakfast cereal samples collected from US retail markets over a 2-year period and found that 205 samples (42%) were OTA positive, but only 16 (3.3%) of oat-based samples showed OTA levels higher than the regulation limit (3 ng/g) of the European Commission. The highest incidence of OTA was in oat-based breakfast cereals (70%), followed by wheat-based (32%), corn-based (15%) and rice-based breakfast cereals (15%) [88]. A more complete assessment conducted in Canada analyzed 2444 finished grain-based product samples collected from commercial markets nationwide, including 55 of baked products, 253 of baking mixes, 330 of beers, 954 of breakfast cereals, 433 of breads, 102 of cookies, 157 of crackers and 160 of pastas. This assessment found 60–84 samples were OTA positive, but only 9 samples contain OTA higher than the EU regulation limit of 3 µg/kg 85]. However, it was found in the same study that among 627 cereal-based infant food samples, 144 of them exceeded the EU limit of 0.5 µg/kg [85]. Another study also reported higher OTA contents (0.6–22.1 ng/g) in 47 (30%) of 155 cereal-based infant foods available in the US market [87]. A recent exposure and risk assessment study of OTA in the US indicates that there was no significant link between dietary exposure to OTA and the risk of adverse effects in the US population with the exception of infants and young children because the OTA exposure was highest in infants and young children who consume large amounts of oat-based cereals [110]. In the Netherlands, cereals were found to be the main contributors (55%) to the total OTA intake. It was estimated that the 99th percentile of the lifelong averaged intake of OTA was 28 ng/kg bw/week, which was considerably lower than the provisional tolerable intake of 100 ng/kg bw/week. Thus, the dietary intake of OTA in the Netherlands should pose no health risk to humans [111].

#### 4.1.3. Fumonisins in Cereal-Based Foods

Human exposure to fumonisins is usually low in Europe and North America as shown in Table 3. High exposures to FB_1_ were reported in Guatemala, Zimbabwe and China, with a maximum of 7700 ng/kg body weight per day for adults living in one rural province of China, while the highest mean exposures for total fumonisins were reported in Malawi, ranging from 3000 to 15,000 ng/kg bw per day [45]. In Italy, the highest fumonisin levels were recorded in puffed (extruded) corn with FB_1_ and FB_2_ levels up to 6100 ng/g and 520 ng/g. The levels of these two fumonisins in corn grits and corn flour/polenta were 420–3760 ng/g FB_1_ and 80–910 ng/g FB_2_, respectively. All sweet corn samples examined were positive for FB_1_ at levels from 60 to 790 ng/g, but negative for FB_2_. Lower levels of fumonisins were found in popcorn (up to 60 ng/g FB_1_ and 20 ng/g FB_2_), tortilla chips (up to 60 ng/g FB_1_ and 10 ng/g FB_2_) and corn flakes (10 ng/g FB_1_) [112]. Although these findings indicate a relatively higher degree of human exposure to fumonisins in Italy among European countries through corn-based food products [112], it is much lower than that in Africa and China. 

#### 4.1.4. Trichothecenes in Cereal-Based Foods

Among trichothecenes, the most important are HT-2 toxin and T-2 toxin from type A and DON from type B. In a study conducted in Spain, DON was found in all types of cereal-based food items with a low prevalence in beer (1.4%), sweet corn (2.8%) and sliced bread (16.7%), and a high prevalence in breakfast cereals (74.1 and 73.4%), corn snacks (78.9%), pasta (74.3%) and bread (100%). Despite the high incidence, only five samples exceeded the EU limits of DON. The HT2 and T-2 toxins were present in low percentages and low contents [113]. A systematic review and meta-analysis of 57 published research/survey articles found that the DON concentration and prevalence in cereal-based foods were higher than other trichothecenes, with a few exceptions. The overall order of contamination based on total trichothecenes concentration was breakfast cereals > noddle > bread > wheat foods > pasta > infant foods > barley. In addition, the prevalence of these mycotoxins did not decrease significantly over the years from 2000 to 2019 [114]. 

Another study determined that AFs, FUMs, DON, HT-2 toxin, OTA, T-2 toxin and ZEA were in 215 infant foods and breakfast cereal collected from three regions in the US, and one or more mycotoxins were found in 69% (101/147) of the infant formulas and 50% (34/68) of breakfast cereals; meanwhile, the mycotoxin co-occurrence was observed in 12% of infant foods and 32% of breakfast cereals. However, the concentrations of detected mycotoxins were lower than the current FDA action and guidance levels [115]. The prevalence and concentration of each mycotoxin often vary with the types of food products. A systematic review found that the prevalence of different mycotoxins in the cereal foods was in the order of OTA > DON > ZEA > AF > 15-ADON > 3-ADON, but the concentration of mycotoxins in the cereal foods was in the order of DON > ZEN > 15-ADON > OTA > 3-ADON > AF [116].

#### 4.1.5. Zearalenone in Cereal-Based Foods

The studies up to 2010 about the occurrence of ZEA in human foods including grains, nuts, edible oils, animal tissues, milk, eggs, miscellaneous foods and spices, as well as human exposure to ZEA were reviewed by Maragos [117]. Based on this review, the estimated tolerable intake of ZEA could be in the range of 0.05–0.5 µg/kg bw/day depending on the toxicological endpoint selected (oestrogenic effect, tumorigenicity, etc.). This review also shows that human exposure to ZEA varied from country to country and in many areas, particularly in African and Asian countries, the safe level can be easily exceeded [117]. One study found that the incidence of ZEA in maize-based products for human consumption on the Spanish market was 40–80 and 44% with levels ranging from 34–216 µg/kg, respectively [89]. It was also reported that the percentages of ZEA-positive samples ranging from 40% to 80% with mean values of 3.8 ± 1.8 μg/kg in pasta, 6.3 ± 5.4 μg/kg in wheat flakes, 5.9 ± 6.8 μg/kg in corn snack, 4.9 ± 0.7 μg/kg sweet corn, 3.7 ± 4.5 ug/kg in sliced bread, 3.1 ± 1.4 in beer and 4.1 ± 0.6 μg/kg in baby food in the market of Catalonia, Spain [90]. It is obvious that most corn-based products have more than 10 times higher ZEA contents than wheat-based products. The tolerable ZEA of 0.05–0.5 µg/kg bw/day may be easily exceeded if corn-based products are frequently consumed, which is the case in Africa.

### 4.2. Mycotoxin Contamination of Feed Products

Studies have shown that mycotoxins pose a high risk to animal health. Cereals including maize, wheat, barley, sorghum and oats grains are the most common ingredients of animal feed. They supply most of the nutrients for livestock animals. For example, swine and poultry diets contain a cereal and cereal by-product fraction of up to 50–60% on a dry matter basis [118]. The by-products of oilseed crops such as the residues of soybeans, peanuts, cottonseed, sunflower, sesame and palm after oil extraction are also used as vegetable protein sources in the manufacturing of animal feed [119]. Among the ingredients of animal feeds, corn and oil seed by-products are mostly susceptible to mycotoxin contamination which places animals at higher risk of mycotoxicoses. 

The study of Schiavone et al. found that poultry feed samples collected from ten poultry farms in Italy in 2006 were all contaminated with OTA at levels from 0.04 to 6.50 μg/kg [118]. A survey of 2000 samples from 52 countries found that mycotoxin contamination in feeds could be up to 79% or higher [120]. A recent survey investigated the individual and co-occurrence of AFB_1_, DON and ZEA in 2090 feed ingredients and 1417 complete feed samples collected from various provinces of China from 2018 to 2020. The results show that AFB_1_, DON and ZEA were present in 81.9, 96.4 and 96.9% of feed samples with concentration ranges of 1.2–27.4, 458.0–1925.4 and 48.1–326.8 μg/kg, respectively. Notably, the levels of AFB_1_, ZEA and DON in 0.9, 0.5 and 0.1% of feed ingredients, and 1.2–12.8, 0.9–2.9 and 0–8.9% of complete feeds for pigs, poultry and ruminants exceeded China’s safety standards (20, 1000 and 500 μg/kg, respectively). Moreover, more than 81.5% of feed ingredients and 95.7% of complete feeds were co-contaminated with different combinations of these mycotoxins [121].

Biomin Research Center in Australia conducted a large-scale global survey of mycotoxin contamination in feed and quantified concentrations of AFB_1_, ZEA, FUMs, OTA, DON and T-2 toxin in 74,821 samples of feed and feed ingredients (including maize, wheat and soybean) collected from 100 countries from 2008 to 2017. They found that 88% and 64% of the samples were contaminated by at least one mycotoxin and more than 2 mycotoxins, respectively, with the most frequently observed mycotoxin combinations being DON, ZEA and FUMs, or FUMs and AFB_1_ [122]. Although in most countries the majority of samples met the regulation limits of the EU, 41.1, 38.5 and 20.9% of feed samples from South Asia, Southeast Asia, and sub-Saharan Africa, respectively, exceeded the maximum allowed level for AFB_1_ (20 µg/kg). There was a distinct regional trend and an obvious year-to-year variation that could be explained by rainfall or temperature during critical stages of crop growth [122]. FB_1_ was also found in 62.5% of samples of maize-based products for human consumption and 100% of corn samples used to make pet food in the Brazilian state of São Paulo [112]. Therefore, livestock animals and pets are often exposed to feeds containing higher levels of mycotoxins.

## 5. Evidence of Mycotoxin Impacts on Human Health

### 5.1. Acute Mycotoxicosis in Human

#### 5.1.1. Acute aflatoxicosis in human

Acute mycotoxin poisoning appears as outbreaks that are mostly caused by aflatoxins. The symptoms of aflatoxicosis include edema, convulsions, vomiting, jaundice, abdominal pain, sudden liver failure and, lastly, death [123]. In humans, acute toxicity due to exposure to high dietary doses of AFs (2000–6000 μg/day) was first reported in Western India in 1974, which led to 106 deaths—a 10% fatality. The outbreak was traced to corn heavily contaminated with *A. flavus* and containing up to 15 mg/kg AFs [10]. In 1981, Kenya experienced its first recorded aflatoxin outbreak with 20 patients aged 2.5 to 45 years, and 12 of them eventually died due to hepatic failure developed within 1 to 12 days following hospital admission [11]. Another outbreak of acute aflatoxicosis in Kenya in 2004 took more than 125 lives [12]. Thereafter, two smaller outbreaks occurred in Kenya: one in 2005, and one in 2006, which resulted in another 53 deaths [31]. The most recent human aflatoxin poisoning was reported in the United Republic of Tanzania in 2016 with a total of 68 cases and 20 of them died. The homegrown maize contaminated with high levels of aflatoxins (10–51,100 μg/kg) was considered to be responsible for the outbreak. In addition to aflatoxins, 8 of 10 maize samples were also contaminated with high levels of fumonisins (945–12,630 μg/kg) [13]. In 1967, twenty-six people in Taiwan were victims of the consumption of moldy rice containing about 200 μg/kg of Afs, and three of them died [124]. In 1988, thirteen Chinese children lost their lives in a city in northwestern Malaysia due to acute hepatic encephalopathy caused by consuming Chinese noodles contaminated with high-level AFs hours prior to their death [125]. 

#### 5.1.2. Acute Mycotoxicoses in Human-Caused by Fusarium Mycotoxins

Exposure to high dosages of DON through consuming highly contaminated grains may induce gastroenteritis, emesis, and a shock-like condition and display vomiting symptoms. Furthermore, an elevated incidence of upper respiratory tract infection was also reported in children who ate wheat bread containing DON for longer than 7 days [126]. T-2 toxin is the most potent among trichothecene toxins. Dietary exposure to T-2 toxin can cause serious health problems such as irritation, hemorrhage, and also necrosis of the gastrointestinal tract (GIT), although the incidence of T-2 toxin poisoning is rare. The T-2 toxin is believed to be responsible for the alimentary toxic aleukia outbreaks which took thousands of lives in Russia [127]. Oral gavage of FB_1_ resulted in toxic effects on different organs including the liver, lung, kidney, heart and intestine in different animals, reduced the cellular activity of immune cell lymphocytes and caused thymocyte apoptosis [128]. The obvious toxic effects such as loss of feed intake and lameness but not lethal effects were observed for young male rats at oral FB_1_ doses of 21.5 and 46.4 mg/kg bw [129]. In female mice, the oral and intraperitoneal LD_50_ values of DON were estimated to be 78 mg/kg and 49 mg/kg, respectively, while the LD_50_ values of 15Ac-DON were 34 mg/kg and 113 mg/kg. At the acute doses, these toxins caused extensive necrosis of the GI tract, bone marrow and lymphoid tissues, and focal lesions in kidney and cardiac tissue [130]. The acute toxicity of ZEA is relatively low compared to other mycotoxins. The oral LD_50_ values of ZEA for mice and rats were reported to be 2000 (male)–20,000 (female) mg/kg bw and 4000 (male)–10,000 (female) mg/kg bw, respectively, with higher toxicity to male [131]. Both animal studies and in vitro studies discovered that the acute toxicity of FB_1_ was much lower than other mycotoxins [131,132].

#### 5.1.3. Acute Ochratoxicosis in Humans

The in vivo acute toxicities of ochratoxins have not been directly studied using a human model. Instead, animals with some biological similarities to humans, such as mice, rats and pigs, were used. Animal studies show that the oral lethal dose LD_50_ of mycotoxins varies with animal species, age and sex. The oral LD_50_ values of OTA 38–56 mg/kg body weight (bw), depending on the animal species with dogs and pigs being more sensitive to OTA than rats and mice [131]. OTA resulted in 30% and 90% mortality in mice at 20 and 50 mg/kg bw, respectively [21,35]. 

In addition to animal studies, in vitro cytotoxicity studies using different human cell lines have also been applied to predict the in vivo acute and subacute toxicity of individual mycotoxins and synergistic or antagonistic effects of different mycotoxins by determining the inhibitory concentrations causing 20, 50 and 80% cell death (IC_20_, IC_50_ and IC_80_) [132,133], to reveal their pathogenic mechanisms (such as oxidative stress, inhibition of translation, DNA damage, apoptosis and signaling pathway in host cells [126,127,134].

### 5.2. Chronic Health Impact of Mycotoxins in Human

Mycotoxins present many threats in chronic conditions to human health. The effect caused by mycotoxicosis related to human health depends on multiple factors such as age, weight, gender/sex, type and quantity of food consumed, contact with infectious agents, and the existence of other types of mycotoxins and bioactive substances. Exposure to small quantities of aflatoxins by oral, respiratory, or absorption by the skin can cause, for example, cancer, liver diseases, teratogenic and genetic mutations [135]. A recent review points out that AFs and FBs are the most relevant mycotoxins, resulting in recognized adverse effects in fetuses and children. Exposure to AFs during embryo development is associated with fetal growth retardation, while exposure to FUMs increases the risk of neural tube defects in newborn babies [134]. Infants are exposed to mycotoxins through breast milk (AFM_1_), con infant formulas and baby foods containing mycotoxin-contaminated ingredients such as animal milk, rice, oat and soybean protein [134].

#### 5.2.1. Chronic Health Impact of Aflatoxins

It has been known for decades that chronic exposure to AFs causes liver cancer in humans and several animal species [136]. Studies have found that people who consumed food contaminated with AFs were related to liver cancer development [135,136]. Moreover, people with hepatitis B and C, which are common diseases in Africa and China, had a higher risk of liver cancer than people who did not have hepatitis when exposed to aflatoxins [14,137]. It is estimated that 40% (59,900 of the 155,000) global annual cases of aflatoxin-induced liver cancer occur in Africa [138]. Liver cancer is also one of the most common cancers with high mortality in China. Maize as the main food staple may significantly contribute to the high incidence of liver cancer in these areas. For example, the population living in the Qigong area of China, where maize was the staple food crop, had a higher incidence of liver cancer before 1989. A government-facilitated change of dietary staple from maize to rice, which is low in or may have aflatoxins, resulted in decreased median levels of the aflatoxin biomarker in serum samples from 19.3 pg/mg albumin in 1989 to undetectable (<0.5 pg/mg) in 2009, and a 65% reduction of liver cancer mortality [139]. A systematic review of epidemiological studies including 13 case–control studies and one longitude study confirmed the positive association between the consumption of aflatoxin-contaminated foods and primary liver cancer risk [140]. In addition to liver cancer, AFs are reported to have teratogenic and mutagenic effects in humans and animals, even at low concentrations, although many aspects of the mechanisms of aflatoxin toxicity remain to be elucidated [33,141]. AFs have been shown to be mutagenic and genotoxic in bacteria, and have the potential to cause birth defects in children and suppress immune function, thus decreasing resistance to infectious agents such as HIV and tuberculosis, as indicated in a few studies conducted in Gambian and Ghana [45,142]. 

#### 5.2.2. Chronic Health Impact of Ochratoxin A

The toxicities of OTA include genotoxicity, carcinogenicity, nephrotoxicity, hepatotoxicity, teratogenicity and immunotoxicity based on in vitro and animal studies. The mode of action of OTA seems to be very complex and is not clearly understood yet. The possible toxigenic mechanisms may include inhibition of protein synthesis and energy production, induction of oxidative stress, DNA adduct formation, as well as apoptosis/necrosis and cell cycle arrest [36]. OTA has been found to be teratogenic in several animal models including rats, mice, hamsters, quail, and chicken, with reduced birth weight and congenital disabilities being the most common symptoms [143]. Cell studies have shown that OTA impairs cellular antioxidant defense responses by regulating the nuclear factor erythroid 2-related factor 2 (NFE2 L2)-mediated pathway, activating ERK- and JNK/MAPK-mediated pathways and NADPH oxidation which triggers the ROS-mediated programmed cell death [127]. However, the potential of OTA to cause malformations in humans and its teratogenic mode of action are unknown. Thereby, more studies in this area are needed. 

OTA has been suspected as a cause of various human nephropathies such as Balkan endemic nephropathy (BEN) and associated urinary tract tumors (UTT), and chronic interstitial nephropathy (CIN) in Tunisia [144,145] since the 1970s because OTA was found more frequently and/or in higher concentration in food and blood of residents in the BEN regions than in other regions [36,146,147] although the involvement of OTA in the development of BEN is inconclusive. BEN is a unique familial, chronic renal disease encountered with a high-prevalence rate in Serbia, Bulgaria, Romania, Croatia and Bosnia and Herzegovina and the affected individuals develop kidney damage that slowly progresses over 10 to 20 years to kidney failure [148]. Significantly higher OTA concentrations in serum or plasma have been found in patients with certain kidney disorders in Bulgaria, Romania, Spain, the Czech Republic, Turkey, Italy, Egypt, Algeria and Tunisia, although OTA may not be the only cause of the diseases [149,150]. A preliminary study in Egypt also found a possible correlation between OTA and renal disease because a high OTA level was found in the serum of end-stage renal disease patients and urothelial cancer patients [151].

The OTA and its metabolite are excreted in urine, but OTA is also found in serum due to its long elimination half-life of about 35 days [38]. Therefore, many studies have used serum OTA and urine OTA as biomarkers of human OTA exposure. In a study in the UK, the urine-OTA level was found to be a better indicator of OTA consumption than the plasma-OTA level [150]. In Bulgaria, a much higher prevalence of OTA (exceeding 2 µg/L) was observed in the blood of the affected population and more frequently in the urine of people living in BEN-endemic villages [152]. In a recent study conducted in the Czech Republic, OTA, CIT and its metabolite DH-CIT were frequently detected in the urine and blood samples of patients with malignant renal tumors (OTA 62%; CIT 91%; DH-CIT 100%) whose urine OTA, CIT and DH-CIT concentrations were in the ranges of 1–27.8 ng/L, 2–87 ng/L and 2–160 ng/L, respectively, and blood OTA levels were 40–870 ng OTA/L serum and 21–182 ng CIT/L plasma [153]. More evidence of the nephrotoxicity of OTA to human beings can be found in a recent review [154] and is not repeated here. In addition to kidney-related diseases, the mouse and human epidemiological studies found OTA as a potential risk factor for human neural tube defects among populations with long-term consumption of fumonisin-contaminated diet, particularly maize [134,155]. Higher OTA concentrations were also detected in the plasma samples of young children with digestive, autism spectrum and attention deficit hyperactivity disorders [134].

#### 5.2.3. Chronic Health Impact of Fumonisins

Structurally, fumonisins are similar to the sphingolipids, including sphingoid bases, sphinganine and sphingosine. FB_1_ is neurotoxic, hepatotoxic and nephrotoxic in animals, and it has been classified as a possible carcinogen to humans. Cell culture studies have revealed that fumonisins disrupt sphingolipid metabolism, folate transport and neural tube development in embryo culture. At the cell level, FB_1_ induces oxidative stress, apoptosis and cytotoxicity, as well as alterations in cytokine expression [156]. In addition, fumonisins can also cause damage to organs such as the kidneys and liver [15,157]. There is sufficient evidence for the carcinogenicity of fumonisins in experimental animals but not in humans; the FB_1_ is thus classified as a possible carcinogen (Group 2B) by IARC [24]. The in vitro and laboratory animal studies suggest an additive or synergistic effect between fumonisin and aflatoxin on the development of precancerous lesions or liver cancer, but currently, there are few studies to support co-exposure as a contributing factor in human disease [45]. The toxicity of OTA and FB_1_ could be enhanced when both toxins are ingested due to the synergy between these two toxins [132].

Epidemiological studies performed in Asia and South Africa between 1988 and 2018 revealed a linkage between esophageal/liver cancers and dietary fumonisin exposure [16,17]. Some studies have shown high FB_1_ content in the diets of particular areas [16,17,22]. An early study analyzed 31 corn samples collected from households in the counties of Cixian and Linxian of China, where high incidences of esophageal cancer have been reported, and they found high levels of FB_1_ (18–155 mg/kg; mean, 74 mg/kg) and total type-A trichothecenes (139–2030 µg/kg; mean, 627 µg/kg) in 16 of the samples, but low levels of aflatoxins in all samples (1–38.4 µg/kg; mean, 8.61 µg/kg) [22]. Another study investigated the co-contamination of AFB_1_ and FB_1_ in 209 food samples in three different areas including Huaian, Fusui and Huantai in China. The results showed higher AFB_1_ (13.5 µg/kg) and FB_1_ (2.6 mg/kg) in corn samples collected from Huaian where the incidence of esophageal cancer was high, with the highest median level of AFB_1_ in cooking oil in Fusui (52.3 µg/kg) where the residents had a high risk of liver cancer, and low AFB_1_ and FB in food items in Huantai where the risks of both oesophageal and liver cancers were low. Based on measured food consumption data, the average daily dietary intake of AFB_1_ per resident was low inHuantai 0.397 but high in Huaian (1.723 µg) and Fusui (2.685 µg), while the average FB_1_ daily dietary intake in these three areas was 92.4, 460.0 and 138.6 µg, respectively [19]. A recent case–control study also revealed that mycotoxin exposure, especially to AFB_1_ and FB_1_, was associated with the risk of esophageal squamous cell carcinoma (ESCC), and there was a synergy between co-exposures to these two mycotoxins which might contribute to the increased risk of ESCC in Huaian where corn flour was the staple food [16]. These studies suggest that FB_1_ was associated with esophageal cancer, while AFB_1_ was associated with liver cancer, and the co-exposure to AFB_1_ and FB_1_ in residents of rural China may contribute to the etiology of human chronic diseases in high-risk areas. Interestingly, China regulates the fumonisin content in animal feed but not in human foods.

However, the case–control study using data from two cohorts conducted in China’s Haimen city and Linxian did not find a statistically significant correlation between dietary exposure to FB_1_ and hepatocellular carcinoma (HCC) in the populations of the cities after adjusting for hepatitis B virus infection and other factors. The pooled meta-analysis of these Chinese cohorts also did not show a significant correlation between FB_1_ and HCC [46]. Therefore, more well-designed studies are needed to provide solid evidence about the carcinogenicity of fumonisins in humans.

#### 5.2.4. Chronic Health Impact of Trichothecene Mycotoxin

Both in vitro and in vivo studies found that exposure to trichothecene mycotoxins can activate apoptosis and/or necrosis of cells in many organs including lymphoid, hematopoietic tissues, liver, bone marrow, thymus and GI system, resulting in leukopenia, vomiting and diarrhea that can be lethal [48]. The toxicodynamics of trichothecenes include inhibition of protein synthesis, immunomodulation immunosuppression and genotoxic effects [158]. Very little information is available relating to their toxic properties in humans, but the diet represents an important source of human exposure to trichothecenes [158]. Chronic exposure to a low dosage of DON may result in anorexia, reduced weight gain, fluctuation in growth hormone and abnormal IgA production [125,159]. Chronic exposure to T-2 toxin slows down the cell regeneration of the bone marrow and spleen, weakens the immune system, and causes malfunction of the reproductive system [125]. The effects of NIV on human health have not been reported.

#### 5.2.5. Chronic Health Impact of Zearalenone

Zearalenone has a structural analogy to estrogen. It is known to have four active metabolites/derivatives, including α-zearalenol (α-ZOL), β-zearalenol (β-ZOL), α-zearalanol (α-ZAL) and β-zearalanol (β-ZAL) [160]. The estrogenic activity of ZEA and its derivatives has been identified both in vivo and in vitro, but the toxicity of ZEA and its compounds is more than the estrogenic activity. For instance, oxidative stress and the harm caused by ZEA may be key mediators of their toxicity [131]. The effects of ZEA on the reproductive system include uterus enlargement, reproductive tract change, reduced fertility, and aberrant levels of progesterone and estradiol. Furthermore, ingestion of ZEA during pregnancy decreased the fetal size and survival percentage of embryos [161]. In addition, in vitro studies have illustrated that the ZEA increases the production of reactive oxygen species (ROS) and, consequently, oxidative stress which play a critical role in ZEA genotoxicity including causing DNA damage, dysregulating DNA repair mechanisms, changing epigenome of targeted cells and affecting chromatin conformation and non-coding RNA [162,163]. 

## 6. Impact of Mycotoxins on Livestock Animals 

Mycotoxins produce a wide range of harmful effects in animals. The economic impact of mycotoxins due to reduced animal productivity, increased incidence of disease (due to immunosuppression and damage to important organs) and decreased reproductive capacity is many times higher than the impact on animal death. Mycotoxins impair the functions of different organs and tissues at lower concentrations, including the digestive system, kidney or liver tissue and the neurological, reproductive and immune systems [164]. Given the wide range of feedstuffs used and the variations between and within animals, the severity of mycotoxicosis from feed differs in many animal species [5]. For example, monogastrics are sensitive to trichothecenes, while poultry and ruminants appear to be less sensitive to some trichothecenes [50]. Poultry is also adversely affected by both T-2 and DON but is very resistant to the estrogenic effects of ZEA [165]. 

Although the symptoms of aflatoxicoses vary with the animal species, some symptoms are common in all animals. Aflatoxin can cause liver damage, decreased reproductive performance, reduced milk or egg production, embryonic death, teratogenicity (birth defects), tumors and suppressed immune system function, even at low oral doses [166]. Unweaned animals may be affected by exposure to AFM_1_ and other aflatoxin metabolites secreted in the milk, while weaning and weaned young animals are most susceptible to the effects of aflatoxin, although all ages are affected. The clinical signs include gastrointestinal dysfunction, reduced productivity, decreased feed utilization and efficiency, anemia and jaundice. OTA has been found to be teratogenic in several animal models including rats, mice, hamsters, quail, and chicks, with reduced birth weight and craniofacial abnormalities being the most common signs. The presence of OTA also results in congenital defects in the fetus [143].

### 6.1. Ruminants 

Ruminants including mammals, goats/sheep, and deer and cattle are often considered less sensitive to mycotoxins owing to rumen microflora converting mycotoxins to less toxic compounds [167]. However, if ruminants consume food infected with mycotoxins for long periods, their development (milk, meat, or wool), fertility, and growth can be disrupted [164]. The contamination of dairy feed with high levels of different mycotoxins has been frequently reported; consequently, clinical signs such as reduced feed intake and feed conversion, reduced milk production and reproduction capacity, lameness, immunosuppression, hepatotoxicity and nephrotoxicity were observed [168,169]. The mycotoxin contamination of ruminant feed is also a hazard to human health since some mycotoxins and their metabolites are excreted in milk, such as AFM_1_ [170], or accumulated in tissues, such as OTA [171,172].

#### 6.1.1. Effects of Aflatoxins on Ruminants

Although feed with low AFs is not considered to be a health risk to ruminants, highly contaminated feed also causes sickness or the death of ruminants, particularly young cattle. Feeding cattle with feed contaminated with aflatoxin at 150–200 µg/kg in (total ration dry matter) TRDM reduced growth and feed efficiency in cattle under 136 kg; reduced growth, feed efficiency and sometimes liver damage in cattle over 136 pounds at 220–400 µg/kg or higher in TRDM; moderate reduction in milk at ≥600 µg/kg or higher in TRDM, pronounced drop (50%) and sharp decrease in milk production if the feed intake at ≥2400 µg/kg in TRDM; deaths in young cattle at ≥ 600 µg/kg or higher in TRDM and in adults at 1000–2000 µg/kg feed in TRDM [173]. Dairy caws exposed to aflatoxin-contaminated feed produce milk with AFM_1_ which is a risk factor to infants and young children. One survey in Europe detected AFB_1_ (1.14 ± 0.10 μg/kg) and AFB_2_ (0.20 ± 0.03 μg/kg) in one of 60 local market milk samples and AFM_1_ in three imported products including condensed milk, milk-based infant formula and table cream at concentrations of 0.10 to 0.40 μg/kg [174]. The analysis of AFM_1_ concentration in 31,702 milk samples in Italy between 2013 and 2018 found that the AFM_1_ concentration in average quality milk was ranging from 9 to 27 ng/kg, and varied with sample collection month and year [175].

#### 6.1.2. Effects of OTA on Ruminants

The sensitivity of ruminants to OTA is lower compared to non-ruminants. The ruminal microbes, with protozoa being a central group, convert OTA extensively into the non-toxic OTα, with OTA disappearance half-lives of 0.6–3.8 h and completely back to 0 after 10–24 h in vivo depending on the OTA dose [176]. As a result, the majority of the OTA and OTAα is excreted into the urine; thus, no OTA residue has been detected in the meat and other tissues of ruminants, although it is occasionally detected in milk at very low concentrations in different studies (0.005–0.058 ng/mL) [176]. According to Zhang and colleagues, the OTA and OTα were distinguishable in urine samples but not in milk and organ tissues of dairy cows, which were given a single dose of feed artificially contaminated with OTA (30 µg/kg bw) [177]. An oral dose of 5–100 μg OTA/kg in cows did not result in residual accumulation of OTA in the liver, kidney, muscles and jejunoileal of the cow as LC-MS/MS was used for quantification [178]. However, contradictory results were obtained in a recent study in which the OTA levels in 120 raw milk samples tested in Egypt-based dairy animals (cow, buffalo, sheep, and goat) exceeded the standard limit (0.5 µg/kg) when OTA concentration was quantified by ELISA, and similar results were found in fresh milk samples in Germany [179]. This could be resulted from the use of ELISA for OTA quantification as ELISA often gives much higher concentration than HPLC or LC-MS/MS.

#### 6.1.3. Effects of Trichothecenes on Ruminants

The available data about the effects of trichothecenes in the ruminant feed are limited, and thus it is difficult to make a science-based risk assessment. The trichothecenes were reported to be mostly transformed to the less toxic de-epoxide metabolite in the rumen before absorption in early studies of trichothecenes metabolism. Additionally, no effect was found on milk production, feed intake, or other parameters measured at levels used in the studies [180]. However, some recent studies found that fusarium toxins with the exception of fumonisins had negative effects on feed digestibility, immunity, weight gain, milk production and reproductive function of cattle and dairy cows [181].

### 6.2. Pigs

#### 6.2.1. Effects of Aflatoxin on Pigs

As monogastric animals, pigs are more sensitive to mycotoxins than ruminants, especially nursing or nursery-age swine. In general, mycotoxins cause reductions in feed intake, growth performance and immune function in pigs at relatively low levels [166]. Aflatoxins induce lesions in the liver, spleen, lymph node, kidney, uterus, heart and lungs of swine, and acute aflatoxin poisoning causes collapse and death within several hours [182]. The tolerance of aflatoxins in pig diets depends on age. Feeding pigs less than 117-days old with feed containing 170–280 µg/kg in total ration dry mass (TRDM) of aflatoxin caused liver damage, reduced growth and feed efficiency and some deaths at 400–600 ppb in TRDM [173]. Chronic exposure to low levels of dietary AFB_1_ for a long period suppressed growth performance, reduced apparent total tract digestibility and damaged intestinal barrier integrity in pigs [183]. The regulation limits or action levels of aflatoxin are 20 μg/kg for immature swine feed, 100 μg/kg for breeding swine and 200 μg/kg for finishing swine in the US [184]. 

#### 6.2.2. Effects of OTA on Pigs

Swine are more sensitive to OTA than ruminants because the ruminal microbes, with protozoa being the dominant group, can convert OTA into less toxic OTα, but swine cannot [176]. Recent studies found that a subchronic exposure of weaned piglets to OTA at the EU regulation limit (50 μg/kg) for 30 days caused measurable hepatocellular injury and negatively affected the immune response and the antioxidant self-defense at the gut and kidney level of weaned piglets [185,186]. Piglets with a body weight of about 10 kg exposed to a diet artificially contaminated with OTA at a level of 75 μg/kg feed, which is higher than the EU regulation (50 μg/kg) but lower than the regulation limits in many developing countries (100 μg/kg), for 42 days significantly enhanced the replication of porcine circovirus 2 (PCV2) DNA through oxidative stress-mediated p38/ERK1/2 MAPK signaling pathway [187], but the OTA concentration in organs such as liver, kidney, lung and spleen were lower than the regulation limit of foods for human consumption. It also induced nephrotoxicity and immunotoxicity in porcine kidney cells (PK 15) [188]. These recent studies indicate that OTA contamination of cereal grains and feed is a potential health risk to swine, although the pork meat and organs from pigs fed with a contaminated diet have low OTA residue.

#### 6.2.3. Effects of Fumonisins on Pigs

Swine is less sensitive to fumonisins than to other mycotoxins. It was found that exposure to a moderate concentration of fumonisins (11.8 mg/kg feed) in naturally contaminated feed for 63 days did not result in observable health issues in pigs but altered the digestive microbiota balance, especially for *Salmonella*-infected pigs [189]. A recent study also reported that diets containing up to 21.9 mg/kg of fumonisin, which is higher than the US regulation limit of 10 mg/kg, did not dramatically decrease the growth performance of nursery pigs 9 to 28 kg, but it did at 32.7 mg/kg or higher [190]. However, the swine feed contaminated with a high dose of FB_1_ (100 mg/kg) could cause acute porcine pulmonary edema, which is often lethal, even for 3–5 days of short-period exposure [191].

#### 6.2.4. Effects of DON and ZEA on Pigs

The FB_1_ and DON either alone or in combination have a great impact on the GI tract and the immune system of swine. FB_1_ and DON alter the intestinal barrier, impair the immune response and reduce feed intake and weight gain [192]. A study reported that feeding pigs with a diet contaminated with DON (8.6 mg/kg) and ZEA (1.2 mg/kg) for 42 days resulted in a significant decrease in performance of the piglets, increased relative uterus weight and altered serum parameters [193]. Weaning pigs fed a diet containing 1 mg/kg DON and 250 µg/kg ZEA exhibited significantly reduced feed intake, body weight gain and elevated activities of serum enzymes, such as γ-glutamyltransferase (GGT) alanine aminotransferase (ALT) and aspartate aminotransferase (AST), which might reflect damage to organs, particularly liver [194]. Feed hesitation may be followed by swollen vulvas and the involvement of fertility disorders from ZEA and DON in the same ration. DON-contaminated feed is usually intolerable to swine and induces rejection of feed and vomiting [195]. The evidence on the damaging effects of ZEA on mammalian folliculogenesis from early to final oogenesis stages was comprehensively reviewed by Zhang et al. [42]. Such effects include impaired granulosa cell development and follicle steroidogenesis, reduced oocyte nest breakdown, damaged meiotic progression, poor fetal oocyte survival, accelerated primordial follicle activation and enhanced follicle atresia. These phenomena may result in reproductive and non-reproductive problems in domestic animals. Clinical symptoms in the infected pigs involved reluctance to feed, excessive tiredness and fighting, feeder banging, elevated incidences of sows becoming agitated and stepping and lying on piglets, incidents of slobbering soon after eating and diarrhea outbreaks and indications of abdominal pain, and reduced weight gain [196]. The regulation limit of DON in complete swine feed is 1 mg/kg which is only 10–20% of that for cattle feed [184].

In one study by Tiemann and colleagues, prepubertal gilts were fed with diets contaminated with DON (0.21–9.57 mg/kg) and ZEA (4–358 µg/kg) for 35 days. The symptoms of hyperestrogenism or uterotrophic effects were not observed and the inhibition of concanavalin A-stimulation of blood lymphocytes was detected in treatment groups; however, the proliferation rate of splenocytes was decreased significantly (*P* < 0.05) in pigs given the feed with the highest DON/ZEA content. The presence of hemosiderin particles in the spleen sections was verified by transmission electron microscopic examination, indicating spleen dysfunction (hemosiderosis) in the absence of clinical signs in pigs fed a diet containing a large portion of wheat highly contaminated with fusarium toxin [197]. In another study conducted by the same research group, wheat contaminated naturally with DON and ZEA was fed to pregnant Landrace sows for 35 days. On day 110, a cesarean section was carried out, the offspring were killed immediately after birth. The histopathological evaluation of tissues discovered changes in the liver and spleen tissues of sows but not piglets, although liver damage was not detected in the sera of the pregnant sows. Thus, the research team assumed that there are no adverse effects on the liver and spleen of full-term piglets when their mothers consumed diets containing up to 9570 μg/kg of DON and 358 μg/kg of ZEA [198].

The research of Lee’s group found that chronic ingestion of high doses of DON (8 mg/kg) and ZEA (0.8 mg/kg) for 4 weeks altered the immune response and damaged organs in pigs [199,200,201]. Specifically, DON and ZEA exposure decreased body weight, feed intake, feed conversion ratio and serum immunoglobulin G (IgG) and IgM concentrations. The total antioxidant levels significantly decreased in serum and increased in urine samples of both treatment groups [199]. Additionally, increased urine serotonin levels were detected in DON and ZEA-treated groups. Although hematological parameters were not affected, lesions were observed in sections of kidneys from treatment groups [200]. Further, high concentrations of DON and ZEA altered gene expression profiles of the kidney and liver, suppressed the inflammatory response in kidneys and lead to disruption of immune homeostasis and effects on other immune-related processes in the livers of piglets [201].

### 6.3. Poultry

The effects of different mycotoxins on poultry health have been reviewed by many authors [5,202,203]. Some common effects are reduced feed intake, weight gain, feed efficiency, growth performance, immunity and hatchability along with increased mortality, organ damage (mainly kidney and liver), carcinogenicity, teratogenicity and decreased egg production, although the specific effect varies with type and concentration of mycotoxin exposed to, poultry species, age and sex [203]. The results of recent studies about the health effects of diets contaminated with different mycotoxins at various levels on broilers, laying hens and turkeys are summarized in Table 4. Those studies demonstrate that poultry is very sensitive to AFs and OTA, but can tolerate relatively higher doses of fusarium mycotoxins with the exception of T2-toxin compared to pigs.

#### 6.3.1. Effects of Aflatoxin on Poultry

Aflatoxin affects all poultry species. Young poultry, especially ducks and turkeys, are very susceptible to aflatoxicoses. The single dose LD_50_ is 0.3 (mg/kg bw) for ducklings, and 6.0–16.0 (mg/kg bw) for chickens [202]. The regulation limits of AFB_1_ in completed poultry diets vary with poultry species, age and geographical locations/countries. As a general rule, the maximal allowed for growing poultry is a 20 µg/kg diet. However, the content lower than 20 µg/kg may still increase their risk of sickness, decrease their tolerance to stress and bruising and generally make them unthrifty. An aflatoxin-contaminated diet can reduce the stress tolerance of laying hens by weakening the immune system. The dysfunctional immune system can reduce egg size and possibly lower egg production [167,172]. Researchers have found that feeding chickens with feed contaminated with high doses of aflatoxins mixture significantly reduced body weight and increased the weight of organs such as kidneys and livers. Aflatoxins have also been reported to cause an increase in blood urea-N, and decreased serum levels of total protein, albumin and phosphorus [165].

#### 6.3.2. Effects of OTA on Poultry

Ochratoxins are probably the most harmful mycotoxin for poultry. Starter poultry (days 0–21) are very sensitive to ochratoxins, especially, OTA. The ochratoxins suppress feed intake, growth and egg production and have a negative influence on eggshell strength. OTA exposure has also been shown to cause immunosuppression in birds by negatively impacting cellular, humoral and innate immune responses. Furthermore, OTA has the tendency to accumulate in kidneys, liver and meat, as well as in blood serum and, therefore, the OTA residue represents a potential hazard in the human food chain [35]. The early studies of Dwivedi and colleagues revealed the impacts of OTA exposure on poultry kidneys and livers. Increased accumulation of cytoplasmic glycogen in the hepatocytes was observed in the liver and swelling and discoloration of the kidneys have been reported as one of the most consistent lesions, while decreased serum albumin and total protein levels are the most sensitive indicator of ochratoxicosis in chicken [216,217,218,219]. A recent study revealed the impacts of combined AFB_1_ and OTA on broilers. Feeding broilers with a diet containing OTA and AFB_1_ at a total level of 300 µg/kg or higher resulted in significantly lower body weight gain, lower cell-mediated immunity and humoral immunity, higher levels of liver damage enzymes such as alanine, aminotransferase (ALT) and alkaline phosphatase (ALP), and significant reduction (*P* < 0.05) of serum uric acid and cholesterol levels [220]. The OTA can cause gastrointestinal dysbiosis, including increasing intestine permeability, immunity and bacterial translocation, and can eventually lead to gut and other organ impairment [221]. The regulation limit of OTA in formulated feed for poultry is 100 µg/kg in Asian and European countries, and 200 µg/kg in South Africa, but OTA in feed may not be regulated in Australia, New Zealand, and North, Central and South America (https://www.mycotoxins.info/regulations/, accessed on 10 July 2023). 

#### 6.3.3. Effects of Fusarium Mycotoxins on Poultry

Early studies reported that poultry species were less sensitive to fusarium mycotoxins in comparison to other animals. Fumonisins among fusarium mycotoxins, fumonisins at high concentrations of 150–400 mg FB_1_/kg resulted in multifocal hepatic necrosis in chickens, biliary hyperplasia, diffuse hepatocellular hyperplasia, with biliary hyperplasia evident in turkeys [222,223,224]. The low level of fumonisins (18.6 mg FB_1_ + FB_2_/kg feed) in the diet also causes a shift in intestinal microbial composition in broiler chickens by decreasing the abundance of beneficial bacteria and increasing the population of pathogenic bacteria [210]. Toxic effects of trichothecenes include oral lesions, growth retardation, abnormal feathering, decreased egg production and egg shell quality, regression of the bursa of Fabricius, peroxidative changes in the liver, abnormal blood coagulation, leucopenia and proteinemia and immune suppression [167,204,205,225]. While much is yet to be learned, T2 toxin and related compounds are currently thought to be the most potent fusarium mycotoxin for poultry [167]. More recent studies have shown that broiler chickens, turkeys, laying hens and embryonated eggs are significantly affected when birds are fed for an extended period with grains naturally contaminated with relatively low levels of multiple fusariums which is usually the case in the real world [205,206,207,208,209,210,211]. 

It appears that the fumonisins cause toxicity in animals due to the disruption of sphingolipid metabolism by inhibiting ceramide synthase (sphinganine/sphingosine N-acyltransferase) (SA/SO), and thus lead to an increase in tissue concentrations of the sphingolipids sphingosine (SO) and sphinganine (SA) and a change in the SA:SO [215,226,227]. Therefore, it causes growth retardation and defects in the embryos of hamsters, rats, mice and chickens [228]. In poultry, DON and fumonisins also damage the epithelial intestinal barrier of the poultry’s gastrointestinal tract, inhibit protein synthesis, decrease nutrient absorption and predispose them to develop necrotic enteritis; the extended contact contributes to direct cellular damage, resulting in intestinal inflammation and diarrhea [209,210,229,230]. The extended exposure to feed with higher FB_1_ and DON contents also results in higher FB_1_ and DON residues in the breeder eggs, which is associated with low hatching rate and gizzard ulcerations in chicken progenies [211].

### 6.4. Effects of Mycotoxins on Pets

#### 6.4.1. Mycotoxin Contamination of Pet Foods

Mycotoxin contamination in pet food is a serious health threat to pets. Mycotoxicosis in pets causes emotional and economic concerns for pet owners. AFs, OTs, TRIs, ZEA, FUMs and fusaric acid have been found in pet food ingredients and finished products, resulting in both acute toxicity and chronic health problems in pets [231]. The mycotoxins in pet food are mainly from grains used as ingredients, while grain-free pet foods usually do not have detectable mycotoxins [232]. However, pet foods also contain non-grain ingredients such as animal organs [233], which may also contribute to the total mycotoxin contents of pet foods because animal organs often contain higher levels of mycotoxins than muscle [234,235]. One recent study screened 28 mycotoxins in dry pet food (55 dog food and 34 cat food samples) by ultra-high-performance liquid chromatography coupled with high-resolution mass spectrometry (UHPLC-Q-Orbitrap HRMS), and found mycotoxin contamination in 99% of pet food samples and all positive samples showed co-occurrence of multiple mycotoxins (up to 16 analytes per sample) [236]. A study analyzed main mycotoxins in 32 commercial pet dry food products in China’s market and found 96.9% of them were contaminated by at least three different types of mycotoxins with the incidence rates of DON, ZEN, AFB_1_, FB_1_, CIT and BEA being 78.1%, 62.5%, 87.5%, 93.8%, 68.8 and 96.9%, respectively. Furthermore, AFB_1_ concentrations in all AFB_1_ positive samples were in the range of 30.3–242.7 µg/kg, exceeding the EU and US’s maximum limits [237]. The aforementioned studies clearly demonstrate that pets fed with dry foods containing grains are often exposed to unsafe mycotoxin levels globally.

#### 6.4.2. Outbreaks of Pets Mycotoxicoses

The outbreaks of mycotoxicoses in pets were frequently reported in Western countries. Studies have shown that mycotoxin’s effects on pet animals are severe and can result in death. Worldwide major mycotoxin outbreaks in pets that occurred before 2007 are summarized in an early review [238]. In the US, almost all mycotoxin outbreaks in pets are due to high levels of aflatoxin in dry pet food. The outbreak in 1998 resulted in the death of 25 dogs due to the consumption of dry dog food containing aflatoxin at 35–191 µg/kg feed [239]. More than 75 dogs died and hundreds more developed serious liver problems in the US in 2005–2006 outbreaks after swallowing aflatoxins-contaminated pet food with AFB_1_ contents in the range of 223–579 µg/kg [239]. A large aflatoxicosis outbreak in Southern Brazil in 2011 affected 65 dogs, of which 60 died, from 9 different farms after they were fed diets with cooked corn meal contaminated with 1640 to 1770 µg/kg of AFB_1_ [240]. From 30 December 2020 to 21 January 2021, more than 110 pets died and 210 pets were sick after consuming certain brands of pet food manufactured by Midwestern Pet Foods in the US Most of these cases had been officially confirmed as aflatoxin poisoning through laboratory testing or veterinary record review [241]. In spring 2021, the cat food containing T-2 and HT-2 toxins at levels that exceeded EU recommended values caused severe pancytopenia in an increasing number of cats, leading to at least 365 cats died and hundreds sickened in the United Kingdom (UK). The potato flakes in cat food were the source of mycotoxins [242]. 

#### 6.4.3. Recent Recalls of Dry Pet Foods Due to High Aflatoxin Content

On 20 December 2005, the US Food and Drug Administration (FDA) issued a recall for 19 distinct varieties of pet food produced at a single facility in Gaston, South Carolina. A total of 16 batches of pet food were discovered to be contaminated with aflatoxins at levels of 223–579 µg/kg, which were remarkably higher than the regulation limit of 20 µg/kg [239]. High levels of aflatoxin were found in bagged dog food on a grocery store shelf in Iowa in 2013. The products, all manufactured by the Pro-Pet plant in Kansas city, were recalled across eight Midwestern states due to elevated levels of the aflatoxin in the corn used to make the pet food [240]. On 2 September 2020, Sunshine Mills announced a recall of certain pet food products after an unsafe level of aflatoxin was detected in a retail product sample by the Louisiana Department of Agriculture and Forestry to contain [241]. The recent recalls of pet foods in the US due to high levels of mycotoxins are given in Table 5.

The higher mycotoxin concentrations in pet food and relatively higher frequency of fatal pet mycotoxicosis indicate that mycotoxin contamination of pet food is still a serious food safety issue and a challenge in the pet food industry. Aflatoxins are responsible for most of the outbreaks and recalls. Closer monitoring of the mycotoxin contents of cereal ingredients used for pet food production is urgently needed to ensure pet food safety.

## 7. Efforts to Ensure the Safety of Food and Feed Supplies

Tremendous efforts have been taken to ensure food and feed safety and to protect humans, livestock animals and pets from mycotoxicoses. These efforts including the control of mold infection and mold growth in the field, prevention of mold and mycotoxin contamination in post-harvest handling, processing and storage, detoxification of contaminated products and regulations of mycotoxins levels in unprocessed ingredients and processed products. However, no single method or approach can achieve sufficient mold and mycotoxin control no matter in the pre-harvest stage or post-harvest stage. Integrated management strategies are often needed to reduce mycotoxin exposure.

### 7.1. Prevention of Mycotoxins Contamination before and after Harvest

Mycotoxins contamination can occur before and after cereal grains are harvested. Prevention or control of mold contamination and mold growth is the most important strategy to ensure food and feed safety from mycotoxins. 

In the pre-harvest stage, all efforts are aimed to control mold growth. The effect started with the breeding of resistant seeds by genetic engineering, the use of biological methods of seed treatments such as coating seeds and seedlings with plant-derived antifungal peptides or metabolites and biopriming using bacteria such as *Pseudomonas fluorescen* [243]. In the field, the application of good agricultural practices such as selection of fungal-resistant seeds, crop rotation, tillage, fertilization, irrigation, proper use of fungicides and selection of right planting and harvest times all play significant roles in mold control in the field [244,245,246]. The use of bio-fungicides involves different microorganisms, microbial antagonists, or competitors such as a non-toxigenic strain of *Aspergillus flavus*, that effectively suppress the growth of toxic fungi [247,248]. 

In the post-harvest stage, the control of mold and mycotoxin includes the inhibition of mold growth and the detoxification of mycotoxins [243]. Drying and storage conditions are critical in mold and mycotoxin control. The grains have to be sufficiently dried to a safe moisture level before putting in storage bins to prevent mold growth [249,250]; a good and clean storage facility with an efficient aeration system can significantly inhibit mold growth and mycotoxin production [244,246]. The use of hermetic packaging bags is the most suitable approach for the safe storage of cereal grains for farmers; active antifungal packaging which incorporates antifungal agents such as essential oils and organic acid have received more scientific attention against pathogenic fungi [245,250].

### 7.2. Detoxification of Mycotoxin-contaminated Cereal Grains

Once mycotoxins are produced and their levels exceeded the regulation limits, detoxification becomes necessary in order to reduce food loss and reduce environmental contamination by mycotoxin-contaminated agricultural commodities. Pre-cleaning, automatic optical sorting or manual sorting can significantly reduce the mycotoxin levels, and food/feed processing such as milling, cooking, baking, toasting and extrusion can further reduce mycotoxin levels in the products [245]. Other detoxification methods are the use of mycotoxin binders which inhibit the absorption of mycotoxins from entering the bloodstream through the gut, ozone treatment, base treatment with ammonia or hydrated oxide and biological detoxification [38]. The application of some emerging/novel treatment such as volatile bioactive compounds, cold plasma, ionization or radiation treatment before storing also kill/inhibit molds or convert mycotoxins to non-toxic or less toxic compounds [38,245,246]. Biocontrol using living cells and bioactive metabolites, such as enzymes, has been claimed to be highly applicable to the food and feed industries [243]. The efficient microbial species, including non-toxic bacteria, yeasts and fungi, are listed in a recent review [243]. However, the application of emerging technologies is rare in developing countries due to the relatively high cost. There are still challenges in the application of mycotoxin biocontrol because most of the studies were either conducted in a laboratory or a simple system. In addition, the bio-treatments of cereal grains may significantly modify the texture and flavor of grain products which may be unacceptable for humans or domestic animals.

### 7.3. Regulations of Mycotoxins Levels in Cereal Grains 

Mycotoxin regulations of cereal grains play critical roles in protecting human beings and domestic animals from the health risks of mycotoxin exposure. Adoption and implement of mycotoxin regulations for ingredients and final products are the final controls to ensure the safety of food and feed. Many countries have established or adopted regulations to limit exposure to mycotoxins. Over the years, since the discovery of aflatoxins, the list of countries considering mycotoxin regulation has been growing longer for the protection of consumers. By 2003, more than 100 countries have established maximum tolerable levels for aflatoxins in human food [251]. Table 6 and Table 7 present the regulation limits of aflatoxin, DON, FUMs, OTA and ZEA in unprocessed cereal grains for human food and animal feed. All major mycotoxins discussed in this review are regulated in Iran among Mideast countries, but India only regulates aflatoxins and DON in food products and peanut meal [251]. Codex Alimentarius Commission (CAC), the central part of the Joint FAO/WHO Food Standards Program, has set up the maximum levels and associated sampling plans of contaminants and natural toxicants in food and feed to guide international trade [252]. Due to the establishment and enforcement of mycotoxin regulations, the food products for human beings living in Western countries are relatively safe; thus, mycotoxicoses in humans have been rarely reported since the implementation of these regulations. However, in many developing countries, particularly in Africa, the implementation of the regulation is difficult due to food shortage; thus, mycotoxin control has been difficult [159] and outbreaks of human mycotoxicoses have been frequently reported even in the 21st century.

The maximum allowed levels of mycotoxins in cereal grains for humans and animals established by different countries vary greatly, as shown in Table 6 and Table 7. In industrialized countries, the maximum allowed level of each mycotoxin varies with the types of foods/feed (raw or processed) and feeding target (infant, adult, animal species and age). The European Union (EU) has the most strict mycotoxin regulations for both foods and feed in the world, while some countries do not have regulations for certain mycotoxins, and many countries only regulate AFs in food and feed. For example, OTA and ZEA are not regulated in the US and South Africa, while FUMs are not regulated in China and South Africa, although the high levels of FUMs in some areas of China have been reported.

## 8. Conclusions

This review shows that exposure to mycotoxins is unavoidable because the cereal grains which are staples for both food and feed are more or less contaminated globally. Severe mycotoxin contamination makes the food unsuitable for human and animal consumption. In developed countries, mycotoxin contamination of cereal grains is not a major food safety issue to adult human beings due to tight regulations and sufficient food supplies, but it is still a risk factor to infants and young children and it often threads the health and lives of livestock animals and pets. In developing countries, mycotoxin contamination poses significant human health risks due to the insufficient enforcement of food safety regulations and food shortages. Mycotoxin contamination of cereal grains has been and will still be a big challenge to grain producers and food/feed processors. Because cereals can be contaminated by fungi both in the field and after harvest (particularly during storage), it is extremely important to implement both pre-harvest and post-harvest mycotoxin control strategies to reduce the degree of contamination. In many areas/regions of developing countries, where food shortage is common, the development and application of cost-effective detoxification methods to reduce the toxicity of contaminated grains will be very important. Hence, the effects of the emerging prevention and detoxification methods on the nutritional and sensory quality of cereal grains and food/feed products developed from cereal grains also need to be investigated. In addition, the information on mycotoxin toxicity in humans is very limited; thus, more studies regarding the correlation between consuming foods contaminated with low doses of mycotoxins and chronic diseases are needed. Furthermore, since most cereal grains contain multiple mycotoxins, it is necessary to investigate the synergistic effect of co-exposure to more than one mycotoxin. Furthermore, the reported effects of mycotoxins on pet health are usually from the investigations after outbreaks that were caused by the consumption of seriously contaminated pet foods. Thus, the effects of consuming dry pet food containing mycotoxins up to the regulation limits on pet health also deserve to be studied.

## Figures and Tables

**Table 1:** Major mycotoxins and their levels in the cereal grains produced in different regions of the world in 2020.

Region	Major Mycotoxins	Number of Samples Tested	Positive Rate (%)	Median (µg/kg)	Average Level (µg/kg)	Maximum Level (µg/kg)
Europe	Aflatoxin (total)	3711	7	2	6	92
	ZEA	6185	47	28	100	57,147
	DON	6565	60	263	351	11,875
	T-2	4156	32	15	3	1387
	FUM	4187	46	170	645	16,241
	OTA	3666	15	3	9	560
North America	Aflatoxin (total)	1655	4	4	26	482 (corn)
	ZEA	1661	39	120	293	26,466
	DON	1604	75	474	789	43,517
	T-2	1486	2	80	185	3153
	FUM	1655	48	827	2738	66,588
	OTA	1655	3	3	27	750
South and Central America	Aflatoxin (total)	7258	19	3	5	179
	ZEA	6724	46	61	149	43,852
	DON	6134	61	440	736	26,320
	T-2	3474	20	33	41	321
	OTA	6759	71	3	1832	56,000
	FUM	2095	9	1120	7	86
Asia	Aflatoxin (total)	3350	25	9	47	2495
	ZEA	3247	62	44	145	11,786
	DON	3360	71	365	546	17,550
	T-2	2873	7	22	31	169
	OTA	3225	81	501	1316	35,445
	FUM	2892	25	3	12	571
Middle East and North Africa	Aflatoxin (total)	116	7	2	2	5
	ZEA	119	68	34	134	1928
	DON	119	78	225	497	5170
	T-2	109	17	10	11	30
	OTA	119	76	307	769	8586
	FUM	111	12	2	3	7
Africa (without North Africa)	Aflatoxin (total)	1059	7	4	28	1032
	ZEA	1071	44	32	78	3091
	DON	1071	76	331	592	7254
	T-2	1071	0	37	43	74
	OTA	1071	59	142	452	10,368
	Aflatoxin (total)	1059	5	3	9	84

**Table 2:** Co-occurrence of mycotoxins in unprocessed cereal grains.

Country/Region	Mycotoxins Identified	Above EU Limits (%)	References
Africa (corn, n = 20)	86% of maize and peanut samples contained four mycotoxins including AFB_1_, FB_1_, ZEA and OTA.	AFB_1_: 30% of positive samples (>4 µg/kg)	[63]
USA (corn, n = 1828)	7.6% of samples contain AFs (mean concentration 15.2 ppb), 75.7% contain DON (1.6%: >5000 ppb), and 59.7% have FB (10,000 ppb), 43% contain OTA (4.9 ppb), and 3.4% contain ZEA.	AFs: mean = 15.2 µg/kg, Max = 606 µg/kg DON: 1.6% FB: 59.7% ZEA: 25%	[64]
USA (maize samples, n = 90)	Maize samples collected from 10 locations in Michigan state for 2 years. Every sample was contaminated with at least four and six mycotoxins in 2017 and 2018, respectively. Incidence and severity of each mycotoxin varied by year and across locations.	DON: 1.6% of samples exceeded 5000 µg/kg FUMs: 9.6% of grains exceeded 10,000 µg/kg	[65]
Brazil (n = 230, processed rice)	Total of 55.1% of samples contain more than 1 mycotoxin. 17% had AFs and ZEA, 24.2% had AFs and OTA, 6.2% had AFs and citreoviridin (CTV), 4.6% had OTA and CTV, and 3.1% had ZON and CTV, respectively.	AFs: 10–20 µg/kg in 4%, >20 to 30 µg/kg in 2%, and >30.00 µg/kg in 3%.	[66]
Canada (corn, n = 750)	Aflatoxin in 1.1% of samples (4.4 ppb), DON in 41% of samples (282 ppb), FB in 14% (280 ppb), OTA in 2.7% (34 ppb) and ZEA in 4.5%.	N/A	[67]
China (corn n = 520)	93% contains FBs (mean 2528 ppb). 1.0%, 2.7%, 14%, 22%, 44% and 6.0% of the samples were detected with 7, 6, 5, 4, 3 and 2 kinds of mycotoxins, respectively.	N/A	[68,69]
China (72 barley samples and 83 wheat samples)	40 barley (56%) and 35 wheat (42%) samples were mycotoxin positive. Among the positive samples, at least two mycotoxins were detected in 70% of barley samples and 54% of wheat samples.	DON: 6% barley and 6% wheat samples T-2 toxin: 7% barley and 5% wheat samples	[70]
China (338 unprocessed wheat samples)	40 (11.8%), 77 (22.8%), 49 (14.5%) and 41 (12.1%) samples were contaminated with two, three, four and five mycotoxins, respectively. The rate of co-occurrence of fumonisins with other Fusarium toxins was 37.6%.	DON: 44.7–52.4% ZEA: 13.9 AFB_1_: 0.6–2.1%	[71]
Lebanon (durum wheat from two warehouses, n = 300)	23.3–25.3% of samples had AFB_1_ levels >2 μg/kg, respectively. 52.0% and 44.6% of samples had OTA levels of 0.51–9.71 μg/kg, respectively.	AFB_1_: 23.3–25.3% (>2 µg/kg) OTA: 25.33–28.67% (>3 µg/kg)	[72]
Korea (brown rice, millet, sorghum, maize and mixed cereal, n = 5)	FUMs, DON, nivalenol and ZEA were more frequently and simultaneously detected in all cereal grains, and 54% of wheat samples had at least two mycotoxins. AFB_1_ was detected in 1% (brown rice)–9% (millet) of each grain group with mean levels 1.1–5.2 ng/g.	AFB_1_: 4% (>10 µg/kg)	[73]
Nigeria (rice, n = 21)	AFs in all samples at 28–372 μg/kg. OTA, ZEA, DON, FB_1_ and FB_2_ in 66.7, 53.4, 23.8, 14.3 and 4.8% of the samples. Co-occurrence of AFs, OTA and ZEA was very common, and up to five mycotoxins were detected in one sample.	AFB_1_: 100% (28–372 µg/kg) OTA: 66.7% (134–341 μg/kg)	[74]
India (n = 150, maize)	150 freshly harvested maize samples during 2010–2011 and 2011–2012. 28, 20, 58, 23 and 11 were positive for AFB_1_, OTA FB_1_, DON and T-2 toxin, respectively.	AFB_1_: 18.7% (48–58 µg/kg)	[75]
Pakistan (rice, n = 208)	35% of samples were AFs positive and 19% were OTA positive, respectively.	AFB_1_: 19% of positive Total AFs: 24% positive OTA: 14%	[76]
Pakistan (corn, n = 7)	100% of samples were AFs positive and a higher level of AFG_1_ in all maize varieties. OTA was detected in 71% of maize samples at 2.14–214 μg/kg.	Total AFs: 100% (>20 μg/kg) OTA: 52.2% (>5 μg/kg)	[77]
Africa (corn, n = 444)	AFs and FUM co-contamination occurred in 35% of the samples.	AFs: 31.7% FUMs: 1.3% DON: 8.9% ZEA: 3.8% OTA: 4.2%	[78]
Ghana (maize, n = 180)	72.2% of samples were AFT positive with a total AFT 4.27–441.02 µg/kg, in the order of AFB_1_ > AFB_2_ > AFG_1_ > AFG_2_, 57.2% of samples were OTA positive: 4.00–97.51 µg/kg.	AFs: 70.50% > EU limits, 64.44% > Ghana limits OTA: 54.1% > EU limit, 49.9% > Ghana limit	[79]
Kenya (maize, n = 350)	55% of 350 maize samples collected following the 2004 aflatoxicosis had AFs at levels higher than 20 µg/kg. 35% had levels > 100 ppb.	AFs: 55% (>20 µg/kg)	[80]
Kenya (maize, n = 350)	AFB_1_ and FUM were found in 80 and 85% of the samples, respectively. AFB_1_ in 25% and FUM in 48% of samples exceeded EU limits.	AFB_1_: 25% (>5 μg/kg) FUMs: 48% (>2 mg/kg)	[81]
Kenya (milled rice, n = 204)	Sterigmatocystin aflatoxin, citrinin, OTA, fumonisin, diacetoxyscirpenol, HT2, T2 and DON were identified. 3.5% of samples had six toxins in different combinations.	AFs: 13.5%, OTA: 6%; HT2 + T2: 0.5%	[82]
Uganda (n = 105, different grains)	AFs and OTA were detected in 8.3–100% of samples, and co-occurrence of AFs, OTA and DON ranged from 8.3–35.3%, with the highest incidence in sorghum.	N/A	[83]

N/A: represents data not available.

**Table 3:** Occurrence of mycotoxins in cereal-based processed foods.

Country or Region	Type of Foods	Sample Number	Mycotoxins Detected	Concentration Range (μg/kg)	>EU Limits (%)	Method of Detection	Reference
Canada	Cereal-based infant foods	627	OTA positive in 41% of samples, 114 exceeded Canadian regulation	Up to 4.85 with a mean of 0.59 μg/L	23%	LC-MS	[86]
USA	Infant cereals	155	OTA positive: 47 (30%) of 155 infant cereals	0.6–22.1	100% of the positive samples	LC-MS/MS	[87]
USA	Breakfast cereals	489	OTA in 205 samples (42%)	0.1–9.3	3.3%	HPLC-FLD	[88]
Spain	Corn-based foods	25	The incidence of DON, ZEA ZOL and T-2 was 68, 44, 24% and 0.4%, respectively	DON: 29–195 ZEA: 34–216 ZOL: 36–71 T-2: <50	N/A	GC-FID and HPLC	[89]
Spain	Infant foods	60	Aflatoxins in 12 samples (20%)	N/A	10%	HPLC-FLD	[90]
China	Infant foods in the market	820	Low levels of 12 mycotoxins in the following order: DON (55.7%) > ZEA (8.2%) > FB_1_ (3.7%) > OTA (1.1%) > FB_2_ (0.7%)	AFs: not detected ZEA: 0.2–8.8 DON: 1.1–912.3 FB_1_: 242.3 FB_2_: 252.4 OTA: 0.2–3.0	FBs: 0.87%	LC-MS/MS	[91]
Portugal	Breakfast cereals	26	96% of samples containing multiple mycotoxins including AFs, OTA, FUMs, DON, ZEA	AFB_1_: 0–0.13 OTA: 0–0.1 FB_1_ + FB_2_: 0–84 DON: 59–207.8 ZEA: 0.4–5.6	0%	UPLC-FLD GC-MS UPLC-MS/MS	[92]
Spain	Breakfast cereals	72	AFs in 1 sample	Total AFs: 0.5	0%	HPLC-FLD	[93]
Spain	Corn snacks	72	AFs in 1 sample	0.8	0%	HPLC-FLD	[93]
Romania	Wheat-based foods: flour, bread, pasta and biscuit (n = 181)	181	DON and 15Ac-DON were detected in 63% (114) and 5% (9) of all samples	DON: 1.9–1947 15 Ac-DON: 14.2–32.6	DON: 5%	GC-QqQ-MS/MS	[94]
Brazil	Maize snacks	18	FUM: 100% DON: 8% ZEA: 8%	FUMs: 2.9–303.5 DON: 0–40.4 ZEA: 0–54	N/A	LC/MS/MS	[95]
	Breakfast cereals	10	FUMs: 100% DON: 10%	FUMs: 2.7–551.8 DON: 0–120.8			
	Wheat pasta	30	FUM: 13.3% DON: 100% ZEA: 73.3%	FUMs: 0–130 DON: 83.9–860.8 ZEA: 0–205.6			
	Crackers	14	DON: 100% ZEA: 100%	DON: 139–916 ZEA: 26.9–117.6			
Tanzania	Maize-based flour used for feeding children	41	AFs: 32% DON: 44% Fumonisins: 83%	Total AFs: 0.11–386 DON: 57–825 Fumonisins: 63–2284	N/A	validated HPLC-FLD	[96]
Ghana	Infants and young children’s foods derived from cereal	35	71% of samples contained AFB_1_	AFB_1_: 0.18 ± 0.01 to 36.10 ± 0.32	71% (>0.1 μg/kg)	HPLC-FLD	[97]
Ghana	Rice brands	27	Aflatoxins B_1_, B_2_, G_1_ and G_2_	AFB_1_: 65.77–ND AFB_2_: 19.27–0.01 AFG_1_: 1.05–ND AFG_2_: 0.12–ND	29.6% (>10 µg/kg)	HPLC-FLD	[97]
Ghana	Cereal-based food brands	20	AFB_1_, B_2_, G_1_ and G_2_	AFB_1_: 35.46–0.96 AFB_2_: 4.92–0.51 AFG_1_: 6.95–0.27 AFG_2_: 0.82–0.1	33% (>4 µg/kg)	HPLC-FLD	[98]
Ghana	Pasta brands	6	AFB_1_ and B_2_	AFB_1_: 0.930–0.935 AFB_2_: 0.85–0.853	0%	HPLC-FLD	[98]
Iran	Baby food	40	Aflatoxins B_1_, B_2_ and G_2_ in 20% to 60% of samples	AFB_1_: 0.04–0.84 AFB_2_: 0.01–0.08 AFG_2_: 0.007–008	AFB_1_: 30%	HPLC-FLD	[99]
Iran	Rice-based baby food	30	AFB_1_ was detected in 68.7% (33/48) of samples	AFB_1_: 0–15.15	39.6%	HPLC-FLD	[100]
Morocco	Pasta	106	ZEA, DON, HT-2 and T-2 toxins were present in 51.8%, 43.5%, 34.9% and 16% of samples, AFB_1_ in 2 samples	AFB_1_: 0–0.25 ZEA: 0.5–3.0 DON: 16–900 HT-2: 4–419 T-2: 4–50	DON: 21%	LC/MS/MS	[101]
South Africa	Corn-based opaque beers	32	94% of samples had 2–5 mycotoxins AFB_1_: 6%, FB_1_: 53%, FB_2_: 32%, FB_3_: 6%, DON: 84%	AFB_1_: 5.8–7.0 Total FBs: 36–182 DON: up to 72	AFB_1_: 6%	LC-MS	[102]
Namibian	Sorghum malt *omalodu*	45	AFB_1_, AFB_2_ and AFG_1_ in 14%, 5% and 3% of *otombo* malts. FB_1_, FB_2_ and FB_3,_ in 42%, 22% and 3% of *otombo* malts, respectively	AFB_1_: 0.61–28.3 AFB_2_: 0.14–2.35 AFG_1_: 0.39–6.95 FB_1_: 12–500.2 FB_2_: 7.55–79.46 FB_3_: 21.6–136.6	AFB_1_: 20% (>5 µg/kg)	LC/MS/MS	[102]
Namibian	Sorghum malt *otombo*	36	Aflatoxin B_1_, B_2_ and G_1_ in 14%, 5% and 3% of *otombo* malts Fumonisin B_1_, B_2_ and B_3_ in 42%, 22% and 3% of *otombo* malts, respectively	AFB_1_: 0.56–54.2 AFB_2_: 0.5–4.48 AFG_1_: 0.4 FB_1_: 8.17–88.3 FB_2_: 5.92–46.8 FB_3_: 22	AFB_1_: 40% (>5 µg/kg)	LC/MS/MS	[103]
Pakistan	Processed foods	125	38% of 125 samples were contaminated with four types of aflatoxins	AFB_1_: 0.02–1.24 AFB_2_: 0.02–0.37 AFG1: 0.25–2.7 AFG2: 0.21–1.3		HPLC-FLD	[104]
Pakistan	Breakfast cereal	237	41%: AFs OTA: 48% ZEA: 53%	AFB_1_: LOD ^‡^—6.90 Total AFs: LOD—7.45 OTA: LOD—8.45 ZEA: LOD—118.10	AFB_1_: 16% Total AFs: 8% OTA: 30% ZEA: 8%	HPLC-FLD	[105]
Pakistan	Wheat products Spaghetti Noodles Macaroni Lasagne Bucatini	25 34 29 37 22	36, 24, 34, 24 and 36% of spaghetti, noodles, macaroni, lasagne and bucatini were AFs positive, and 28, 18, 34, 32 and 50% were ZEA positive, respectively	AFs: LOD—55.6 ZEA: LOD—69.8	AFs: 18–28% ZEA: 15–36%	HPLC-FLD	[106]

^‡^ LOD—limit of detection.

**Table 4:** Recent studies of impacts of fusarium mycotoxins contaminated feed on poultry health.

Poultry Species, Age and Feeding Period	Sample Size (n)	Mycotoxins in Diet and Concentrations	Health Effects	References
Broiler chicken, 1 day old, fed for 56 days	360	Diet contaminated with fusarium mycotoxins: 0.14–9.7 mg/kg DON, 18–21.6 mg/kg fusaric acid (FA), 0.1–0.8 mg/kg ZEA	Body weight gain and feed intake of chickens decreased quadratically; blood erythrocyte count and serum uric acid concentration increased linearly and the serum lipase activity decreased linearly; a significant quadratic effect on serum albumin and γ-glutamyltransferase activity; blood hemoglobin and biliary IgA concentrations responded in significant linear and quadratic patterns. Efficiency of feed utilization was not affected.	[204]
Broiler chicken, 1 day old, fed for 42 days	360	Fusarium mycotoxin, 5.9–9.5 mg/kg DON, 19.1–21.4 mg/kg fusaric acid (FA), 0.4–0.7 mg/kg ZEA and 0.3–0.5 mg/kg 15AC-DON	Body weight gains and feed intake of chickens decreased linearly while peripheral blood monocytes decreased linearly with increasing toxin levels during the grower stage (21–42 days). Reduced B-cell count linearly but increased the T-cell count on day 28.	[205]
Turkey, 1 day old (n = 300), fed for 12 weeks	300	Blends of grains naturally contaminated with fusarium mycotoxins: DON, 15Ac-DON ZEA and FA	Turkey’s performance and some blood and immunological parameters were adversely affected by feedborne fusarium mycotoxins, and polymeric glucomannan mycotoxin adsorbent (GMA) prevented most of the adverse effects.	[206]
Male broilers at 7 d of age, fed for 5 weeks	75	Diets contain 0.265, 1.68 and 12.2 mg of DON/kg; 0.013, 0.145 and 1.094 mg ZEA/kg	The weekly weight gain decreased linearly (*P* ≤ 0.041) with increasing DON levels during the first 3 weeks of exposure; the weight gain was not influenced thereafter. As the levels of DON increased, the titers against Newcastle disease virus increased linearly during week 2 and week 4 of exposure, but decreased linearly (*P* = 0.006) during week 5 of exposure.	[207]
25-wk-old laying hens (n = 384)	384	Birds were fed diets contaminated with AFB and DON for a 6-wk phase followed by a 4-week recovery phase	Elevated relative liver and kidney weights (*P* < 0.05), reduced feed intake, egg production and egg weights (*P* < 0.05) at the medium and high toxin levels following the toxin phase, but the deactivation compound reduced (*P* < 0.05) relative liver and kidney weights following the recovery period.	[208]
One-day-old broiler chicks (n= 308), fed for 16 days	308	Diet contaminated by FBs (18.6 mg FB_1_ + FB_2_/kg feed)	A significant increase in the plasma sphinganine/sphingosine ratio. Villus height and crypt depth of ileum were significantly reduced. Changed the microbiota composition in the ileum. A higher percentage of chickens fed an FB-contaminated diet developed subclinical necrotic enteritis following *C. perfringens* challenge.	[209]
One-day-old broiler chicks, fed for 15 days	308	Diet 1: contaminated with 4.6 mg DON/kg. Diet 2: contaminated with 25.4 mg FB_1_ + FB_2_/kg. Diet 3: containing 4.3 mg DON and 22.9 mg FB_1_ + FB_2_/kg.	Changed the intestinal mucus layer and several intestinal epithelial antioxidative mechanisms. Both mycotoxins decreased gene expression of the intestinal zinc transporter (ZnT)-1 and regulated intracellular methionine homeostasis, which are both important for preserving the cell’s critical antioxidant activity.	[210]
SPF embryonated eggs aged 11 days	221	Inoculated into albumen with different doses of FB_1_, FB_2_ or DON, or their combinations.	Reduced hatching rate, caused gizzard ulcers and hemorrhagic lungs. Resulted in higher mortality of the progeny of breeder hens and higher mycotoxin residues in the gizzards and the lungs of the progenies.	[211]
5-day-old chickens, fed for 16 weeks	280	Diets containing 0, 2, 5 and 10 mg/kg of DON.	The diets contaminated with DON at 5 mg/kg caused heavier spleens, increase DON-induced cellular proliferation, apoptosis and DNA damage signals in the spleen. Expression of gatekeeper protein claudin-5 was increased in jejunum of female birds but decreased in that of male birds.	[212]
Broiler chickens	70	Five groups of chickens fed control diets (mycotoxin free), the DON diets (5 mg/kg), the FB diet (20 mg FB_1_ + FB_2_/kg), ZEA diet (0.5 mg/kg) and diets contained 5, 20, and 0.5 mg/kg of DON, FB_1_ + FB_2_, and ZEA.	No difference in performances between groups that could be attributed to FBs, the relative weight of organs, biochemistry, histopathology, intestinal morphometry, indicators of oxidative damage and markers of testicle toxicity. Significantly increased sphinganine and SA to SO ratio in broilers fed FB-added diets.	[213]
Turkeys of 55-day old fed experimental diet for 14 days	70	Five groups of turkeys fed with control diets, the DON diets (5 mg/kg), the FB diet (20 mg FB_1_ + FB_2_/kg), ZEA diet (0.5 mg/kg) and diets with DON, FB_1_ + FB_2_, and ZEA (5, 20, and 0.5 mg/kg of), respectively.	Increased the SA to SO ratio in the liver of turkeys fed diets containing FB, but had no apparent toxication symptoms. No interactions/synergies among DON, FB, and ZON.	[214]
One-day-old chicks used for each trial (n = 2200)	2200	Feed contaminated with low levels of mycotoxins (below EU’s regulatory limits).	A strong positive relationship was observed between broilers’ feed efficiency and DON (R^2^ = 0.85), FBs (R^2^ = 0.53), DAS (R^2^ = 0.86), ZEA (R^2^ = 0.92), ENNs (R^2^ = 0.60) and BEV (R^2^ = 0.73). The mixture of ZEA, DON and FBs (*p* = 0.01, R^2^ = 0.84), and the mixture of ZEA, DON and DAS (*p* = 0.001, R^2^ = 0.91) had significant interactive effect on the birds’ feed efficiency.	[215]
Day-old male cobb chicks (1600 birds, 64 pens, 25 birds/pen)	1600	Broilers were fed control diet with minimal mycotoxins and formulated diet containing moderate levels of fusarium mycotoxins (MT).	Compared with control, the diet containing moderate fusarium mycotoxins reduced body weight (BW), increased feed efficiency on days 35 and 42 with increased duodenal crypt depth and reduced goblet cells, and poultry production efficiency.	[64]

**Table 5:** Recalls of pet foods in the US due to mycotoxin contamination since 2019 [241].

Date of Recall	Brand Name	Product Description	Recall Reason	Company Name
July 29, 2021	Triumph, Evolve, Nature Farms, Elm and others	Dog Food	High levels of aflatoxin	Sunshine Mills, Inc., Halifax, VA, USA
January 11, 2021	Sportmix, Nunn Better, ProPac and others	Dog and Cat Pet Food	Aflatoxin exceeds acceptable levels	Midwest Pet Food, Inc., Evansville, IN, USA
December 30, 2020	Sportmix	Dog and Cat Food	High levels of aflatoxin	Midwest Pet Food, Inc., Evansville, IN, USA
October 8, 2020	Champ, Field Trial, Good Dog and others	Pet Food	Possibly aflatoxin	Sunshine Mills, Inc., Halifax, VA, USA
September 2, 2020	Family Pet, Heartland Farms and Paws Happy Life	Dog Food	Elevated levels of aflatoxin	Sunshine Mills, Inc., Halifax, VA, USA
September 23, 2019	Gramco	Hog Grower Pellets	High levels of vomitoxin (DON)	Gramco, Inc., Springville, NY, USA
May 6, 2019	Southern States	Various Animal Feed	Elevated aflatoxin levels	Cargill, Inc., Cleveland, NC, USA

**Table 6:** Maximum allowed levels of major mycotoxins in cereal grains for human foods.

		European Union (µg/kg)	CAC (µg/kg)	US (µg/kg)	Brazil (µg/kg)	China (µg/kg)	India (µg/kg)	South Africa (µg/kg)
Total Aflatoxins	Total	4–10	15	20	50	20	15	10
Aflatoxin B_1_	B_1_	2–5	10	20	30	5–20	10	5
Deoxynivalenol (DON)		1250–1750	2000	1000	200–3000	1000	1000	NR
Fumonisins (B_1_ + B_2_)	B_1_ + B_2_	1000–4000 *	4000	2000–4000 *	1000–5000 *	NR	NR	NR
Ochratoxin A		3–5	5	NR	50	5	NR	NR
Zearalenone (ZEA)		100–350 *	100	NR	100–600 *	60	NR	NR
Reference		[253]	[252]	[251]	[254]	[255]	[256]	[251]

* The regulation limit of a specific mycotoxin varies with the food products to be made using the grain. NR—not regulated.

**Table 7:** Maximum allowed levels of major mycotoxins in cereal grains for livestock animal feed in different countries.

		European Union (µg/kg)	US (µg/kg)	Brazil (µg/kg)	China (µg/kg)	India (µg/kg)	South Africa (µg/kg)
Total Aflatoxins	Total	50	20–300 *	50		NR	
Aflatoxin B_1_	B_1_	20		NR	10–50 *	NR	50
Deoxynivalenol (DON)		8000	5000–10,000 *	NR	1000–5000 *	NR	5000
Fumonisins (B_1_ + B_2_)		60,000	5000–100,000 *	NR	5000–50,000 *	NR	50,000
Ochratoxin A		250	NR	NR	100	NR	200
Zearalenone (ZEA)		2000–3000	NR	NR	100–500 *	NR	5000
Reference		[253]	[251]	[253,254]	[257]	[256]	[253,258]

* The concentration of specific mycotoxin in the grain varies with animal species and age. NR—not regulated.

## Data Availability

Not applicable.

## References

[1] Malachová A., Stránská M., Václavíková M., Elliott C.T., Black C., Meneely J., Hajšlová J., Ezekiel C.N., Schuhmacher R., Krska R. (2018). Advanced LC–MS-based methods to study the co-occurrence and metabolization of multiple mycotoxins in cereals and cereal-based food. Anal. Bioanal. Chem..

[2] Freire L., Sant’ana A.S. (2018). Modified mycotoxins: An updated review on their formation, detection, occurrence, and toxic effects. Food Chem. Toxicol..

[3] Pitt J., Taniwaki M.H., Cole M. (2013). Mycotoxin production in major crops as influenced by growing, harvesting, storage and processing, with emphasis on the achievement of Food Safety Objectives. Food Control.

[4] Patriarca A., Pinto V.F. (2017). Prevalence of mycotoxins in foods and decontamination. Curr. Opin. Food Sci..

[5] Zain M.E. (2011). Impact of mycotoxins on humans and animals. J. Saudi Chem. Soc..

[6] Yogendrarajah P., Jacxsens L., De Saeger S., De Meulenaer B. (2014). Co-occurrence of multiple mycotoxins in dry chilli (*Capsicum annum* L.) samples from the markets of Sri Lanka and Belgium. Food Control.

[7] Neme K., Mohammed A. (2017). Mycotoxin occurrence in grains and the role of postharvest management as a mitigation strategies. A review. Food Control.

[8] De Ruyck K., De Boevre M., Huybrechts I., De Saeger S. (2015). Dietary mycotoxins, co-exposure, and carcinogenesis in humans: Short review. Mutat. Res.-Rev. Mutat. Res..

[9] Reddy B.N., Raghavender C.R. (2007). Outbreaks of Aflatoxicoses in India. Afr. J. Food Agric. Nutr. Dev..

[10] Obura A., Unnevehr L.J., Grace D. (2013). Aflatoxicosis: Evidence from Kenya. Aflatoxins: Finding Solutions for Improved Food Safety.

[11] Probst C., Njapau H., Cotty P.J. (2007). Outbreak of an Acute Aflatoxicosis in Kenya in 2004: Identification of the Causal Agent. Appl. Environ. Microbiol..

[12] Kamala A., Shirima C., Jani B., Bakari M., Sillo H., Rusibamayila N., De Saeger S., Kimanya M., Gong Y., Simba A. (2018). Outbreak of an acute aflatoxicosis in Tanzania during 2016. World Mycotoxin J..

[13] Marasas W.F., Kellerman T.S., Gelderblom W.C., Coetzer J.A., Thiel P.G., Van Der Lugt J.J. (1988). Leukoencephalomalacia in a horse induced by fumonisin B1 isolated from Fusarium moniliforme. Onderstepoort J. Veter.-Res..

[14] Wu Q., Dohnal V., Kuca K., Yuan Z. (2013). Trichothecenes: Structure-Toxic Activity Relationships. Curr. Drug Metab..

[15] Rheeder J., Marasas W., Theil P., Sydenham E., Shephard G., Van Schalkwyk D. (1992). Fusarium moniliformeand Fumonisins in Corn in Relation to Human Esophageal Cancer in Transkei. Phytopathology.

[16] Xue K.S., Tang L., Sun G., Wang S., Hu X., Wang J.-S. (2019). Mycotoxin exposure is associated with increased risk of esophageal squamous cell carcinoma in Huaian area, China. BMC Cancer.

[17] Chen C., Riley R.T., Wu F. (2018). Dietary Fumonisin and Growth Impairment in Children and Animals: A Review. Compr. Rev. Food Sci. Food Saf..

[18] Sun G., Wang S., Hu X., Su J., Huang T., Yu J., Tang L., Gao W., Wang J.S. (2007). Fumonisin B1 contamination of home-grown corn in high-risk areas for esophageal and liver cancer in China. Food Addit. Contam..

[19] Sun G., Wang S., Hu X., Su J., Zhang Y., Xie Y., Zhang H., Tang L., Wang J.S. (2011). Co-contamination of aflatoxin B1 and fumonisin B1 in food and human dietary exposure in three areas of China. Food Addit. Contam..

[20] Marasas W.F.O. (1995). Fumonisins: Their implications for human and animal health. Nat. Toxins.

[21] Gelderblom W.C.A., Semple E., Marasas W.F.O., Farber E. (1992). The cancer-initiating potential of the fumonisin B mycotoxins. Carcinogenesis.

[22] Chu F.S., Guo Y. (1994). Simultaneous occurrence of Fumonisin B1 and other mycotoxins in moldy corn collected from the People’s Republic of China in regions with high incidences of esophageal cancer. Appl. Environ. Microbiol..

[23] Alizadeh A.M., Roshandel G., Roudbarmohammadi S., Roudbary M., Sohanaki H., Ghiasian S.A., Taherkhani A., Semnani S., Aghasi M. (2012). Fumonisin B1 Contamination of Cereals and Risk of Esophageal Cancer in a High Risk Area in Northeastern Iran. Asian Pac. J. Cancer Prev..

[24] IARC (2012). Monographs on the evaluation of carcinogenic risks to humans: Chemical agents and related occupations. A Review of Human Carcinogens.

[25] Wild C.P. (2007). Aflatoxin Exposure in Developing Countries: The Critical Interface of Agriculture and Health. Food Nutr. Bull..

[26] Ropejko K., Twarużek M. (2021). Zearalenone and Its Metabolites—General Overview, Occurrence, and Toxicity. Toxins.

[27] Ferrigo D., Raiola A., Causin R. (2016). Fusarium Toxins in Cereals: Occurrence, Legislation, Factors Promoting the Appearance and Their Management. Molecules.

[28] Dohlman E., Jean C., Buzby J.C. (2003). Mycotoxin hazards and regulations: Impacts on food and animal feed crop trade. International Trade and Food Safety/AER-828.

[29] van Veen T.W.S. (2005). International trade and food safety in developing countries. Food Control.

[30] Kumar P., Mahato D.K., Kamle M., Mohanta T.K., Kang S.G. (2017). Aflatoxins: A Global Concern for Food Safety, Human Health and Their Management. Front. Microbiol..

[31] Schmale D.G., Munkvold G.P. (2009). Mycotoxins in Crops: A Threat to Human and Domestic Animal Health. Plant Health Instr..

[32] Ülger T.G., Uçar A., Çakıroğlu F.P., Yilmaz S. (2020). Genotoxic effects of mycotoxins. Toxicon.

[33] Theumer M., Henneb Y., Khoury L., Snini S., Tadrist S., Canlet C., Puel O., Oswald I., Audebert M. (2018). Genotoxicity of aflatoxins and their precursors in human cells. Toxicol. Lett..

[34] Kuiper-Goodman T., Scott P.M. (1989). Risk assessment of the mycotoxin ochratoxin A. Biomed. Environ. Sci. BES.

[35] Heussner A.H., Bingle L.E.H. (2015). Comparative Ochratoxin Toxicity: A Review of the Available Data. Toxins.

[36] Kőszegi T., Poór M., Manderville R.A., Pfohl-Leszkowicz A. (2016). Ochratoxin A: Molecular Interactions, Mechanisms of Toxicity and Prevention at the Molecular Level. Toxins.

[37] Fuchs R., Peraica M. (2005). Ochratoxin A in human kidney diseases. Food Addit. Contam..

[38] Agriopoulou S., Stamatelopoulou E., Varzakas T. (2020). Advances in Occurrence, Importance, and Mycotoxin Control Strategies: Prevention and Detoxification in Foods. Foods.

[39] Gupta R.C., Srivastava A., Lall R., Gupta R.C. (2018). Chapter 72—Ochratoxins and Citrinin. Veterinary Toxicology.

[40] Longobardi C., Ferrara G., Andretta E., Montagnaro S., Damiano S., Ciarcia R. (2022). Ochratoxin A and Kidney Oxidative Stress: The Role of Nutraceuticals in Veterinary Medicine—A Review. Toxins.

[41] Lonkar P., Dedon P.C. (2010). Reactive species and DNA damage in chronic inflammation: Reconciling chemical mechanisms and biological fates. Int. J. Cancer.

[42] Zhang G.-L., Feng Y.-L., Song J.-L., Zhou X.-S. (2018). Zearalenone: A Mycotoxin with Different Toxic Effect in Domestic and Laboratory Animals’ Granulosa Cells. Front. Genet..

[43] Rogowska A., Pomastowski P., Sagandykova G., Buszewski B. (2019). Zearalenone and its metabolites: Effect on human health, metabolism and neutralisation methods. Toxicon.

[44] Shier W.T., Shier A.C., Xie W., Mirocha C.J. (2001). Structure-activity relationships for human estrogenic activity in zearalenone mycotoxins. Toxicon Off. J. Int. Soc. Toxinol..

[45] WHO (2018). World Health Organization Food Safety Digest—Fumonisins. https://www.who.int/foodsafety/FSDigest_Fumonisins_EN.pdf.

[46] Persson E.C., Sewram V., Evans A.A., London W.T., Volkwyn Y., Shen Y.-J., Van Zyl J.A., Chen G., Lin W., Shephard G.S. (2012). Fumonisin B1 and risk of hepatocellular carcinoma in two Chinese cohorts. Food Chem. Toxicol..

[47] McCormick S.P., Stanley A.M., Stover N.A., Alexander N.J. (2011). Trichothecenes: From Simple to Complex Mycotoxins. Toxins.

[48] Janik E., Niemcewicz M., Podogrocki M., Ceremuga M., Stela M., Bijak M. (2021). T-2 Toxin—The Most Toxic Trichothecene Mycotoxin: Metabolism, Toxicity, and Decontamination Strategies. Molecules.

[49] Jakovac-Strajn B., Tavčar-Kalcher G. (2012). A Method Using Gas Chromatography—Mass Spectrometry for the Detection of Mycotoxins from Trichothecene Groups A and B in Grains. Gas Chromatography in Plant Science, Wine Technology, Toxicology and Some Specific Applications.

[50] Polak-Śliwińska M., Paszczyk B. (2021). Trichothecenes in Food and Feed, Relevance to Human and Animal Health and Methods of Detection: A Systematic Review. Molecules.

[51] Audenaert K., Vanheule A., Höfte M., Haesaert G. (2014). Deoxynivalenol: A Major Player in the Multifaceted Response of Fusarium to Its Environment. Toxins.

[52] Foroud N.A., Baines D., Gagkaeva T.Y., Thakor N., Badea A., Steiner B., Bürstmayr M., Bürstmayr H. (2019). Trichothecenes in Cereal Grains—An Update. Toxins.

[53] Broekaert N., Devreese M., Demeyere K., Berthiller F., Michlmayr H., Varga E., Adam G., Meyer E., Croubels S. (2016). Comparative in vitro cytotoxicity of modified deoxynivalenol on porcine intestinal epithelial cells. Food Chem. Toxicol..

[54] Paterson R.R.M., Lima N. (2010). How will climate change affect mycotoxins in food?. Food Res. Int..

[55] Streit E., Naehrer K., Rodrigues I., Schatzmayr G. (2013). Mycotoxin occurrence in feed and feed raw materials worldwide: Long-term analysis with special focus on Europe and Asia. J. Sci. Food Agric..

[56] CAST (1989). Mycotoxins: Economics and Health Risks.

[57] Eskola M., Kos G., Elliott C.T., Hajslova J., Mayar S., Krska R. (2020). Worldwide contamination of food-crops with mycotoxins: Validity of the widely cited ‘FAO estimate’ of 25%. Crit. Rev. Food Sci. Nutr..

[58] Biomin (2020). Biomin World Mycotoxin Survey 2020, Annual Report No. 17. https://www.biomin.net/science-hub/world-mycotoxin-survey-impact-2021/.

[59] Bhat R., Rai R.V., Karim A. (2010). Mycotoxins in Food and Feed: Present Status and Future Concerns. Compr. Rev. Food Sci. Food Saf..

[60] Ostry V., Malir F., Toman J., Grosse Y. (2017). Mycotoxins as human carcinogens—The IARC Monographs classification. Mycotoxin Res..

[61] FAO/JECFA (2016). Safety Evaluation of Certain Contaminants in Food.

[62] WHO (2017). Evaluation of Certain Contaminants in Food: Eighty-Third Report of the Joint FAO/WHO Expert Committee on Food Additives.

[63] Sangare-Tigori B., Moukha S., Kouadio H.J., Betbeder A.-M., Dano D.S., Creppy E.E. (2007). Co-occurrence of aflatoxin B1, fumonisin B1, ochratoxin A and zearalenone in cereals and peanuts from Côte d’Ivoire. Food Addit. Contam..

[64] Weaver A.C., King W.D., Verax M., Fox U., Kudupoje M.B., Mathis G., Lumpkins B., Yiannikouris A. (2020). Impact of Chronic Levels of Naturally Multi-Contaminated Feed with *Fusarium* Mycotoxins on Broiler Chickens and Evaluation of the Mitigation Properties of Different Titers of Yeast Cell Wall Extract. Toxins.

[65] Fusilier K., Chilvers M.I., Limay-Rios V., Singh M.P. (2022). Mycotoxin Co-Occurrence in Michigan Harvested Maize Grain. Toxins.

[66] Almeida M.I., Almeida N.G., Carvalho K.L., Gonçalves G.A.A., Silva C.N., Santos E.A., Garcia J.C., Vargas E.A. (2012). Co-occurrence of aflatoxins B_1_, B_2_, G_1_and G_2_, ochratoxin A, zearalenone, deoxynivalenol, and citreoviridin in rice in Brazil. Food Addit. Contam. Part A.

[67] CFIA (2020). Ochratoxin A in Wheat Products, Oat Products, Rice Products and Other Grain Products—1 April 2018 to 31 March 2019. Canadian Food Inspection Agency. https://inspection.canada.ca/food-safety-for-industry/food-chemistry-and-microbiology/food-safety-testing-bulletin-and-reports.

[68] Wang Y., Dong Y.J., Yue H., Li Z.M., Chen Y.B., Wang Y.T., Deng L.G., Zhao S.C. (2016). Investigation and analysis on mycotoxins contamination of maize in Shandong Province. Sci. Technol. Cereals Oils Foods.

[69] Sun X., Su P., Shan H. (2017). Mycotoxin contamination of maize in China. Compr. Rev. Food Sci. Food Saf..

[70] Shi H., Schwab W., Yu P. (2019). Natural Occurrence and Co-Contamination of Twelve Mycotoxins in Industry-Submitted Cool-Season Cereal Grains Grown under a Low Heat Unit Climate Condition. Toxins.

[71] Zhao J., Cheng T., Xu W., Han X., Zhang J., Zhang H., Wang C., Fanning S., Li F. (2021). Natural co-occurrence of multi-mycotoxins in unprocessed wheat grains from China. Food Control.

[72] Joubrane K., Mnayer D., El Khoury A., El Khoury A., Awad E. (2020). Co-Occurrence of Aflatoxin B1 and Ochratoxin A in Lebanese Stored Wheat. J. Food Prot..

[73] Kim D.-H., Hong S.-Y., Kang J.W., Cho S.M., Lee K.R., An T.K., Lee C., Chung S.H. (2017). Simultaneous Determination of Multi-Mycotoxins in Cereal Grains Collected from South Korea by LC/MS/MS. Toxins.

[74] Makun H.A., Dutton M.F., Njobeh P.B., Mwanza M., Kabiru A.Y. (2011). Natural multi-occurrence of mycotoxins in rice from Niger State, Nigeria. Mycotoxin Res..

[75] Mudili V., Siddaih C.N., Nagesh M., Garapati P., Kumar K.N., Murali H.S., Yli-Mattila T., Batra H.V. (2014). Mould incidence and mycotoxin contamination in freshly harvested maize kernels originated from India. J. Sci. Food Agric..

[76] Iqbal S.Z., Asi M.R., Hanif U., Zuber M., Jinap S. (2016). The presence of aflatoxins and ochratoxin A in rice and rice products; and evaluation of dietary intake. Food Chem..

[77] Wajih Ul Hassan S., Sadef Y., Hussain S., Asi M.R., Ashraf M.Y., Anwar S., Malik A. (2020). Unusual pattern of aflatoxins and ochratoxin in commercially grown maize varieties of Pakistan. Toxicon.

[78] Gruber-Dorninger C., Jenkins T., Schatzmayr G. (2018). Multi-mycotoxin screening of feed and feed raw materials from Africa. World Mycotoxin J..

[79] Kortei N.K., Annan T., Kyei-Baffour V., Essuman E.K., Okyere H., Tettey C.O. (2021). Exposure and risk characterizations of ochratoxins A and aflatoxins through maize (*Zea mays*) consumed in different agro-ecological zones of Ghana. Sci. Rep..

[80] Lewis L., Onsongo M., Njapau H., Schurz-Rogers H., Luber G., Kieszak S., Nyamongo J., Backer L., Dahiye A.M., Misore A. (2005). Aflatoxin Contamination of Commercial Maize Products during an Outbreak of Acute Aflatoxicosis in Eastern and Central Kenya. Environ. Health Perspect..

[81] Ngure F., Ngure C., Achieng G., Munga F., Moran Z., Stafstrom W., Nelson R. (2021). Mycotoxins contamination of market maize and the potential of density sorting in reducing exposure in unregulated food systems in Kenya. World Mycotoxin J..

[82] Mutiga S.K., Mutuku J.M., Koskei V., Gitau J.K., Ng’ang’a F., Musyoka J., Chemining’wa G.N., Murori R. (2021). Multiple Mycotoxins in Kenyan Rice. Toxins.

[83] Echodu R., Malinga G.M., Kaducu J.M., Ovuga E., Haesaert G. (2019). Prevalence of aflatoxin, ochratoxin and deoxynivalenol in cereal grains in northern Uganda: Implication for food safety and health. Toxicol. Rep..

[84] Karlovsky P., Suman M., Berthiller F., De Meester J., Eisenbrand G., Perrin I., Oswald I.P., Speijers G., Chiodini A., Recker T. (2016). Impact of food processing and detoxification treatments on mycotoxin contamination. Mycotoxin Res..

[85] Habschied K., Šarić G.K., Krstanović V., Mastanjević K. (2021). Mycotoxins—Biomonitoring and Human Exposure. Toxins.

[86] Kolakowski B., O’Rourke S.M., Bietlot H.P., Kurz K., Aweryn B. (2016). Ochratoxin A Concentrations in a Variety of Grain-Based and Non–Grain-Based Foods on the Canadian Retail Market from 2009 to 2014. J. Food Prot..

[87] Cappozzo J., Jackson L., Lee H.J., Zhou W., Al-Taher F., Zweigenbaum J., Ryu D. (2017). Occurrence of Ochratoxin A in Infant Foods in the United States. J. Food Prot..

[88] Nguyen K.T.N., Ryu D. (2014). Concentration of ochratoxin A in breakfast cereals and snacks consumed in the United States. Food Control.

[89] Cerveró M.C., Castillo M.A., Montes R., Hernández E. (2007). Determination of trichothecenes, zearalenone and zearalenols in commercially available corn-based foods in Spain. Rev. Iberoam. Micol..

[90] Cano-Sancho G., Marin S., Ramos A., Sanchis V. (2012). Occurrence of zearalenone, an oestrogenic mycotoxin, in Catalonia (Spain) and exposure assessment. Food Chem. Toxicol..

[91] Ji X., Xiao Y., Wang W., Lyu W., Wang X., Li Y., Deng T., Yang H. (2022). Mycotoxins in cereal-based infant foods marketed in China: Occurrence and risk assessment. Food Control.

[92] Martins C., Assunção R., Cunha S.C., Fernandes J.O., Jager A., Petta T., Oliveira C.A., Alvito P. (2018). Assessment of multiple mycotoxins in breakfast cereals available in the Portuguese market. Food Chem..

[93] Cano-Sancho G., Sanchis V., Marín S., Ramos A. (2013). Occurrence and exposure assessment of aflatoxins in Catalonia (Spain). Food Chem. Toxicol..

[94] Stanciu O., Juan C., Miere D., Berrada H., Loghin F., Mañes J. (2018). First study on trichothecene and zearalenone exposure of the Romanian population through wheat-based products consumption. Food Chem. Toxicol..

[95] Andrade P.D., Dias J.V., Souza D.M., Brito A.P., van Donkersgoed G., Pizzutti I.R., Caldas E.D. (2020). Mycotoxins in cereals and cereal-based products: Incidence and probabilistic dietary risk assessment for the Brazilian population. Food Chem. Toxicol..

[96] Kimanya M.E., Shirima C.P., Magoha H., Shewiyo D.H., De Meulenaer B., Kolsteren P., Gong Y.Y. (2014). Co-exposures of aflatoxins with deoxynivalenol and fumonisins from maize based complementary foods in Rombo, Northern Tanzania. Food Control.

[97] Blankson G.K., Mill-Robertson F.C. (2016). Aflatoxin contamination and exposure in processed cereal-based complementary foods for infants and young children in greater Accra, Ghana. Food Control.

[98] Kortei N.K., Agyekum A.A., Akuamoa F., Baffour V.K., Alidu H.W. (2019). Risk assessment and exposure to levels of naturally occurring aflatoxins in some packaged cereals and cereal based foods consumed in Accra, Ghana. Toxicol. Rep..

[99] Bashiry M., Yazdanpanah H., Sadeghi E., Shokri S., Mirmoghtadaie L., Mortazavian A.M., Mohammadi A., Nematollahi A., Hejazi E., Hosseini H. (2021). Occurrence of Aflatoxins in Commercial Cereal-based Baby Foods in Iran: A Probabilistic Risk Assessment to Health. Iran J. Pharm. Res..

[100] Mottaghianpour E., Nazari F., Mehrasbi M.R., Hosseini M.-J. (2017). Occurrence of aflatoxin B_1_in baby foods marketed in Iran. J. Sci. Food Agric..

[101] Bouafifssa Y., Manyes L., Rahouti M., Mañes J., Berrada H., Zinedine A., Fernández-Franzón M. (2018). Multi-Occurrence of Twenty Mycotoxinsin Pasta and a Risk Assessment in the Moroccan Population. Toxins.

[102] Adekoya I., Obadina A., Adaku C.C., De Boevre M., Okoth S., De Saeger S., Njobeh P. (2018). Mycobiota and co-occurrence of mycotoxins in South African maize-based opaque beer. Int. J. Food Microbiol..

[103] Nafuka S.N., Misihairabgwi J.M., Bock R., Ishola A., Sulyok M., Krska R. (2019). Variation of Fungal Metabolites in Sorghum Malts Used to Prepare Namibian Traditional Fermented Beverages *Omalodu* and *Otombo*. Toxins.

[104] Mushtaq M., Sultana B., Anwar F., Khan M.Z., Ashrafuzzaman M. (2012). Occurrence of Aflatoxins in Selected Processed Foods from Pakistan. Int. J. Mol. Sci..

[105] Iqbal S.Z., Rabbani T., Asi M.R., Jinap S. (2014). Assessment of aflatoxins, ochratoxin A and zearalenone in breakfast cereals. Food Chem..

[106] Iqbal S.Z., Asi M.R., Jinap S., Rashid U. (2014). Detection of aflatoxins and zearalenone contamination in wheat derived products. Food Control.

[107] Luo S., Du H., Kebede H., Liu Y., Xing F. (2021). Contamination status of major mycotoxins in agricultural product and food stuff in Europe. Food Control.

[108] Li X., Ma W., Ma Z., Zhang Q., Li H. (2021). The Occurrence and Contamination Level of Ochratoxin A in Plant and Animal-Derived Food Commodities. Molecules.

[109] Lee H.J., Ryu D. (2015). Significance of Ochratoxin a in Breakfast Cereals from the United States. J. Agric. Food Chem..

[110] Mitchell N.J., Chen C., Palumbo J.D., Bianchini A., Cappozzo J., Stratton J., Ryu D., Wu F. (2017). A risk assessment of dietary Ochratoxin a in the United States. Food Chem. Toxicol..

[111] Bakker M., Pieters M.N. (2022). Risk Assessment of Ochratoxin A in the Netherlands.

[112] Cruz J.V.d.S. (2010). Ocorrência de Aflatoxinas e Fumonisinas em Produtos à Base de Milho e Milho Utilizado Como Ingrediente de Ração Para Animais de Companhia, Comercializados na Região de Pirassununga. Estado de São Paulo. Ph.D. Thesis.

[113] Cano-Sancho G., Valle-Algarra F., Jiménez M., Burdaspal P., Legarda T., Ramos A., Sanchis V., Marín S. (2011). Presence of trichothecenes and co-occurrence in cereal-based food from Catalonia (Spain). Food Control.

[114] Khaneghah A.M., Farhadi A., Nematollahi A., Vasseghian Y., Fakhri Y. (2020). A systematic review and meta-analysis to investigate the concentration and prevalence of trichothecenes in the cereal-based food. Trends Food Sci. Technol..

[115] Zhang K., Flannery B.M., Oles C.J., Adeuya A. (2018). Mycotoxins in infant/toddler foods and breakfast cereals in the US retail market. Food Addit. Contam. Part B.

[116] Khaneghah A.M., Fakhri Y., Raeisi S., Armoon B., Sant’Ana A.S. (2018). Prevalence and concentration of ochratoxin A, zearalenone, deoxynivalenol and total aflatoxin in cereal-based products: A systematic review and meta-analysis. Food Chem. Toxicol..

[117] Maragos C.M. (2010). Zearalenone occurrence and human exposure. World Mycotoxin J..

[118] Schiavone A., Cavallero C., Girotto L., Pozzo L., Antoniazzi S., Cavallarin L. (2008). A survey on the occurrence of ochratoxin a in feeds and sera collected in conventional and organic poultry farms in Northern Italy. Ital. J. Anim. Sci..

[119] Santos Pereira C., Cunha S.C., Fernandes J.O. (2019). Prevalent Mycotoxins in Animal Feed: Occurrence and Analytical Methods. Toxins.

[120] Kovalsky P., Kos G., Nährer K., Schwab C., Jenkins T., Schatzmayr G., Sulyok M., Krska R. (2016). Co-Occurrence of Regulated, Masked and Emerging Mycotoxins and Secondary Metabolites in Finished Feed and Maize—An Extensive Survey. Toxins.

[121] Zhao L., Zhang L., Xu Z., Liu X., Chen L., Dai J., Karrow N.A., Sun L. (2021). Occurrence of Aflatoxin B1, deoxynivalenol and zearalenone in feeds in China during 2018–2020. J. Anim. Sci. Biotechnol..

[122] Gruber-Dorninger C., Jenkins T., Schatzmayr G. (2019). Global Mycotoxin Occurrence in Feed: A Ten-Year Survey. Toxins.

[123] Mwanda O., Otieno C., Omonge E. (2005). Acute aflatoxicosis: Case report. East Afr. Med. J..

[124] Pitt J.I., Semple R.L., Frio A.S., Hicks P.A., Lozare J.V. (1989). An Introduction to Mycotoxins, in Mycotoxin Prevention and Control in Food Grains.

[125] Lye M.S., Ghazali A.A., Mohan J., Alwin N., Nair R.C. (1995). An outbreak of acute hepatic encephalopathy due to severe aflatoxicosis in Malaysia. Am. J. Trop. Med. Hyg..

[126] Ekwomadu T.I., Akinola S.A., Mwanza M. (2021). *Fusarium* Mycotoxins, Their Metabolites (Free, Emerging, and Masked), Food Safety Concerns, and Health Impacts. Int. J. Environ. Res. Public Health.

[127] Dey D.K., Kang J.I., Bajpai V.K., Kim K., Lee H., Sonwal S., Simal-Gandara J., Xiao J., Ali S., Huh Y.S. (2022). Mycotoxins in food and feed: Toxicity, preventive challenges, and advanced detection techniques for associated diseases. Crit. Rev. Food Sci. Nutr..

[128] Chen J., Wen J., Tang Y., Shi J., Mu G., Yan R., Cai J., Long M. (2021). Research Progress on Fumonisin B1 Contamination and Toxicity: A Review. Molecules.

[129] McKean C., Tang L., Tang M., Billam M., Wang Z., Theodorakis C., Kendall R., Wang J.-S. (2006). Comparative acute and combinative toxicity of aflatoxin B1 and fumonisin B1 in animals and human cells. Food Chem. Toxicol..

[130] Forsell J., Jensen R., Tai J.-H., Witt M., Lin W., Pestka J. (1987). Comparison of acute toxicities of deoxynivalenol (vomitoxin) and 15-acetyldeoxynivalenol in the B6C3F1 mouse. Food Chem. Toxicol..

[131] Zinedine A., Soriano J.M., Moltó J.C., Mañes J. (2007). Review on the toxicity, occurrence, metabolism, detoxification, regulations and intake of zearalenone: An oestrogenic mycotoxin. Food Chem. Toxicol..

[132] Wen J., Mu P., Deng Y. (2016). Mycotoxins: Cytotoxicity and biotransformation in animal cells. Toxicol. Res..

[133] Creppy E.E., Chiarappa P., Baudrimont I., Borracci P., Moukha S., Carratù M. (2004). Synergistic effects of fumonisin B1 and ochratoxin a: Are in vitro cytotoxicity data predictive of in vivo acute toxicity?. Toxicology.

[134] Wentzel J.F., Lombard M.J., Du Plessis L.H., Zandberg L. (2017). Evaluation of the cytotoxic properties, gene expression profiles and secondary signalling responses of cultured cells exposed to fumonisin B1, deoxynivalenol and zearalenone mycotoxins. Arch. Toxicol..

[135] Alvito P., Pereira-Da-Silva L. (2022). Mycotoxin Exposure during the First 1000 Days of Life and Its Impact on Children’s Health: A Clinical Overview. Toxins.

[136] Atkins D., Norman J. (1998). Mycotoxins and food safety. Nutr. Food Sci..

[137] Christopher P., Wild J., Miller D., John D., Groopman J.D. (2015). Mycotoxin Control in Low and Middle-Income Countries.

[138] Wagacha J.M., Muthomi J. (2008). Mycotoxin problem in Africa: Current status, implications to food safety and health and possible management strategies. Int. J. Food Microbiol..

[139] Liu Y., Wu F. (2010). Global Burden of Aflatoxin-Induced Hepatocellular Carcinoma: A Risk Assessment. Environ. Health Perspect..

[140] Chen J.-G., Egner P.A., Ng D., Jacobson L.P., Muñoz A., Zhu Y.-R., Qian G.-S., Wu F., Yuan J.-M., Groopman J.D. (2013). Reduced Aflatoxin Exposure Presages Decline in Liver Cancer Mortality in an Endemic Region of China. Cancer Prev. Res..

[141] Claeys L., Romano C., De Ruyck K., Wilson H., Fervers B., Korenjak M., Zavadil J., Gunter M.J., De Saeger S., De Boevre M. (2020). Mycotoxin exposure and human cancer risk: A systematic review of epidemiological studies. Compr. Rev. Food Sci. Food Saf..

[142] Omotayo O.P., Omotayo A.O., Mwanza M., Babalola O.O. (2019). Prevalence of Mycotoxins and Their Consequences on Human Health. Toxicol. Res..

[143] Gong Y.Y., Watson S., Routledge M.N. (2016). Aflatoxin Exposure and Associated Human Health Effects, a Review of Epidemiological Studies. Food Saf..

[144] Malir F., Ostry V., Pfohl-Leszkowicz A., Novotna E. (2014). Ochratoxin A: Developmental and Reproductive Toxicity—An Overview. Birth Defects Res. B Dev. Reprod. Toxicol..

[145] Abid S., Hassen W., Achour A., Skhiri H., Maaroufi K., Ellouz F., Creppy E., Bacha H. (2003). Ochratoxin a and human chronic nephropathy in Tunisia: Is the situation endemic?. Hum. Exp. Toxicol..

[146] Stefanovic V., Toncheva D., Atanasova S., Polenakovic M. (2006). Etiology of Balkan Endemic Nephropathy and Associated Urothelial Cancer. Am. J. Nephrol..

[147] Krogh P., Hald B., Pleština R., Čeović S. (1977). Balkan (endemic) nephropathy and foodborn ochratoxin a: Preliminary results of a survey of foodstuffs. Acta Pathol. Microbiol. Scand. Sect. B Microbiol..

[148] Pavlović M., Plestina R., Krogh P. (1979). Ochratoxin a Contamination of Foodstuffs in an Area with Balkan (Endemic) Nephropathy. Acta Pathol. Microbiol. Scand. Sect. B Microbiol..

[149] Bui-Klimke T.R., Wu F. (2015). Ochratoxin A and Human Health Risk: A Review of the Evidence. Crit. Rev. Food Sci. Nutr..

[150] Pavlović N.M. (2013). Balkan endemic nephropathy—Current status and future perspectives. Clin. Kidney J..

[151] Scott P.M. (2005). Biomarkers of human exposure to ochratoxin A. Food Addit. Contam..

[152] Wafa E.W., Yahya R.S., Sobh M.A., Eraky I., el-Baz M., el-Gayar H.A., Betbeder A.M., Creppy E.E. (1998). Human ochratoxicosis and nephropathy in Egypt: A preliminary study. Hum. Exp. Toxicol..

[153] Studer-Rohr J., Schlatter J., Dietrich D.R. (2000). Intraindividual variation in plasma levels and kinetic parameters of ochratoxin a in humans. Arch. Toxicol..

[154] Castegnaro M., Canadas D., Vrabcheva T., Petkova-Bocharova T., Chernozemsky I.N., Pfohl-Leszkowicz A. (2006). Balkan endemic nephropathy: Role of ochratoxins A through biomarkers. Mol. Nutr. Food Res..

[155] Malir F., Louda M., Ostry V., Toman J., Ali N., Grosse Y., Malirova E., Pacovsky J., Pickova D., Brodak M. (2019). Analyses of biomarkers of exposure to nephrotoxic mycotoxins in a cohort of patients with renal tumours. Mycotoxin Res..

[156] Khoi C.-S., Chen J.-H., Lin T.-Y., Chiang C.-K., Hung K.-Y. (2021). Ochratoxin A-Induced Nephrotoxicity: Up-to-Date Evidence. Int. J. Mol. Sci..

[157] Marasas W.F.O., Riley R.T., Hendricks K.A., Stevens V.L., Sadler T.W., Gelineau-van Waes J., Missmer S.A., Cabrera J., Torres O., Gelderblom W.C.A. (2004). Fumonisins Disrupt Sphingolipid Metabolism, Folate Transport, and Neural Tube Development in Embryo Culture and In Vivo: A Potential Risk Factor for Human Neural Tube Defects among Populations Consuming Fumonisin-Contaminated Maize. J. Nutr..

[158] Stockmann-Juvala H., Savolainen K. (2008). A review of the toxic effects and mechanisms of action of fumonisin B1. Hum. Exp. Toxicol..

[159] Chilaka C.A., Obidiegwu J.E., Chilaka A.C., Atanda O.O., Mally A. (2022). Mycotoxin Regulatory Status in Africa: A Decade of Weak Institutional Efforts. Toxins.

[160] Sudakin D.L. (2003). Trichothecenes in the environment: Relevance to human health. Toxicol. Lett..

[161] Mokubedi S.M., Phoku J.Z., Changwa R.N., Gbashi S., Njobeh P.B. (2019). Analysis of Mycotoxins Contamination in Poultry Feeds Manufactured in Selected Provinces of South Africa Using UHPLC-MS/MS. Toxins.

[162] Zhang Y., Jia Z., Yin S., Shan A., Gao R., Qu Z., Liu M., Nie S. (2014). Toxic Effects of Maternal Zearalenone Exposure on Uterine Capacity and Fetal Development in Gestation Rats. Reprod. Sci..

[163] Tatay E., Espín S., García-Fernández A.-J., Ruiz M.-J. (2017). Oxidative damage and disturbance of antioxidant capacity by zearalenone and its metabolites in human cells. Toxicol. Vitr..

[164] Feng Y.-Q., Zhao A.-H., Wang J.-J., Tian Y., Yan Z.-H., Dri M., Shen W., De Felici M., Li L. (2022). Oxidative stress as a plausible mechanism for zearalenone to induce genome toxicity. Gene.

[165] Pierron A., Alassane-Kpembi I., Oswald I.P. (2016). Impact of mycotoxin on immune response and consequences for pig health. Anim. Nutr..

[166] Wu K., Ren C., Gong Y., Gao X., Rajput S.A., Qi D., Wang S. (2021). The insensitive mechanism of poultry to zearalenone: A review. Anim. Nutr..

[167] Jones F.T., Genter M.B., Hagler W.M., Hansen J.A., Mowrey B.A., Poore M.H., Whitlow L.W. (1994). Understanding and Coping with Effects of Mycotoxins in Livestock Feed and Forage. North Carolina Cooperative Extension Service. https://projects.ncsu.edu/cals/an_sci/extension/animal/nutr/Understanding_mycotoxins.pdf.

[168] Mostrom M.S., Jacobsen B.J. (2011). Ruminant Mycotoxicosis. Veter.-Clin. N. Am. Food Anim. Pract..

[169] Gallo A., Giuberti G., Frisvad J.C., Bertuzzi T., Nielsen K.F. (2015). Review on mycotoxin issues in ruminants: Occurrence in forages, effects of mycotoxin ingestion on health status and animal performance and practical strategies to counteract their negative effects. Toxins.

[170] Kemboi D.C., Ochieng P.E., Antonissen G., Croubels S., Scippo M.-L., Okoth S., Kangethe E.K., Faas J., Doupovec B., Lindahl J.F. (2020). Multi-Mycotoxin Occurrence in Dairy Cattle and Poultry Feeds and Feed Ingredients from Machakos Town, Kenya. Toxins.

[171] Hof H. (2016). Mycotoxins in milk for human nutrition: Cow, sheep and human breast milk. GMS Infect. Dis..

[172] Jouany J.P., Yiannikouris A., Bertin G. (2009). Risk assessment of mycotoxins in ruminants and ruminant products. Options Mediterr. A.

[173] Upadhaya S.D., Park M.A., Ha J.K. (2010). Mycotoxins and Their Biotransformation in the Rumen: A Review. Asian-Australas. J. Anim. Sci..

[174] Adams R.S., Kephart K.B., Ishler V.A., Hutchinson L.J., Roth G.W. (2016). Mold and Mycotoxin Problems in Livestock Feeding. Penn State Extension. https://extension.psu.edu/mold-and-mycotoxin-problems-in-livestock-feedingAntonissen.

[175] Zhang K., Wong J.W., Hayward D.G., Vaclavikova M., Liao C.-D., Trucksess M.W. (2013). Determination of Mycotoxins in Milk-Based Products and Infant Formula Using Stable Isotope Dilution Assay and Liquid Chromatography Tandem Mass Spectrometry. J. Agric. Food Chem..

[176] Serraino A., Bonilauri P., Kerekes K., Farkas Z., Giacometti F., Canever A., Zambrini A.V., Ambrus Á. (2019). Occurrence of Aflatoxin M1 in Raw Milk Marketed in Italy: Exposure Assessment and Risk Characterization. Front. Microbiol..

[177] Mobashar M., Hummel J., Blank R., Südekum K.-H. (2010). Ochratoxin A in Ruminants–A Review on Its Degradation by Gut Microbes and Effects on Animals. Toxins.

[178] Zhang Z., Fan Z., Nie D., Zhao Z., Han Z. (2019). Analysis of the Carry-Over of Ochratoxin A from Feed to Milk, Blood, Urine, and Different Tissues of Dairy Cows Based on the Establishment of a Reliable LC-MS/MS Method. Molecules.

[179] Hashimoto Y., Katsunuma Y., Nunokawa M., Minato H., Yonemochi C. (2016). Influence of repeated ochratoxin A ingestion on milk production and its carry-over into the milk, blood and tissues of lactating cows. Anim. Sci. J..

[180] Turkoglu C., Keyvan E. (2019). Determination of Aflatoxin M1 and Ochratoxin A in Raw, Pasteurized and UHT Milk in Turkey. Acta Sci. Vet..

[181] Eriksen G.S., Pettersson H. (2004). Toxicological evaluation of trichothecenes in animal feed. Anim. Feed. Sci. Technol..

[182] Gallo A., Mosconi M., Trevisi E., Santos R.R. (2022). Adverse Effects of *Fusarium* Toxins in Ruminants: A Review of in Vivo and in Vitro Studies. Dairy.

[183] Popescu R.G., Rădulescu A.L., Georgescu S.E., Dinischiotu A. (2022). Aflatoxins in Feed: Types, Metabolism, Health Consequences in Swine and Mitigation Strategies. Toxins.

[184] NGFA, National Grain and Feed Association (2011). FDA Regulatory Guidance for Mycotoxins—A Guide for Grain Elevators, Feed Manufacturers, Grain Processors and Exporters.

[185] Pu J., Yuan Q., Yan H., Tian G., Chen D., He J., Zheng P., Yu J., Mao X., Huang Z. (2021). Effects of Chronic Exposure to Low Levels of Dietary Aflatoxin B_1_ on Growth Performance, Apparent Total Tract Digestibility and Intestinal Health in Pigs. Animals.

[186] Marin D., Motiu M., Pistol G., Gras M., Israel-Roming F., Calin L., Stancu M., Taranu I. (2016). Diet contaminated with ochratoxin A at the highest level allowed by EU recommendation disturbs liver metabolism in weaned piglets. World Mycotoxin J..

[187] Marin D.E., Pistol G.C., Gras M.A., Palade M.L., Taranu I. (2017). Comparative effect of ochratoxin A on inflammation and oxidative stress parameters in gut and kidney of piglets. Regul. Toxicol. Pharmacol..

[188] Gan F., Zhang Z., Hu Z., Hesketh J., Xue H., Chen X., Hao S., Huang Y., Ezea P.C., Parveen F. (2015). Ochratoxin A promotes porcine circovirus type 2 replication in vitro and in vivo. Free Radic. Biol. Med..

[189] Gan F., Zhou Y., Hou L., Qian G., Chen X., Huang K. (2017). Ochratoxin A induces nephrotoxicity and immunotoxicity through different MAPK signaling pathways in PK15 cells and porcine primary splenocytes. Chemosphere.

[190] Burel C., Tanguy M., Guerre P., Boilletot E., Cariolet R., Queguiner M., Postollec G., Pinton P., Salvat G., Oswald I.P. (2013). Effect of Low Dose of Fumonisins on Pig Health: Immune Status, Intestinal Microbiota and Sensitivity to Salmonella. Toxins.

[191] Rao Z.-X., Tokach M.D., Woodworth J.C., DeRouchey J.M., Goodband R.D., Calderón H.I., Dritz S.S. (2020). Effects of Fumonisin-Contaminated Corn on Growth Performance of 9 to 28 kg Nursery Pigs. Toxins.

[192] Ensley S.M., Radke S.L. (2019). Mycotoxins in Grains and Feeds. Diseases of Swine.

[193] Pierron A., Alassane-Kpembi I., Oswald I.P. (2016). Impact of two mycotoxins deoxynivalenol and fumonisin on pig intestinal health. Porc. Health Manag..

[194] Döll S., Dänicke S. (2011). The Fusarium toxins deoxynivalenol (DON) and zearalenone (ZON) in animal feeding. Prev. Vet. Med..

[195] Cheng Y.-H., Weng C.-F., Chen B.-J., Chang M.-H. (2006). Toxicity of different Fusarium mycotoxins on growth performance, immune responses and efficacy of a mycotoxin degrading enzyme in pigs. Anim. Res..

[196] Ogunade I., Martinez-Tuppia C., Queiroz O., Jiang Y., Drouin P., Wu F., Vyas D., Adesogan A. (2018). Silage review: Mycotoxins in silage: Occurrence, effects, prevention, and mitigation. J. Dairy Sci..

[197] Coppock R.W., Jacobsen B.J. (2009). Mycotoxins in animal and human patients. Toxicol. Ind. Health.

[198] Tiemann U., Brüssow K.-P., Jonas L., Pöhland R., Schneider F., Dänicke S. (2006). Effects of diets with cereal grains contaminated by graded levels of two Fusarium toxins on selected immunological and histological measurements in the spleen of gilts1,2. J. Anim. Sci..

[199] Tiemann U., Brüssow K.-P., Dänicke S., Vanselow J. (2008). Feeding of pregnant sows with mycotoxin-contaminated diets and their non-effect on foetal and maternal hepatic transcription of genes of the insulin-like growth factor system. Food Addit. Contam. Part A.

[200] Reddy K.E., Song J., Lee H.-J., Kim M., Kim D.-W., Jung H.J., Kim B., Lee Y., Yu D., Kim D.-W. (2018). Effects of High Levels of Deoxynivalenol and Zearalenone on Growth Performance, and Hematological and Immunological Parameters in Pigs. Toxins.

[201] Reddy K.E., Lee W., Jeong J.Y., Lee Y., Lee H.-J., Kim M.S., Kim D.-W., Yu D., Cho A., Oh Y.K. (2018). Effects of deoxynivalenol- and zearalenone-contaminated feed on the gene expression profiles in the kidneys of piglets. Asian-Australas. J. Anim. Sci..

[202] Reddy K.E., Jeong J.Y., Lee Y., Lee H.-J., Kim M.S., Kim D.-W., Jung H.J., Choe C., Oh Y.K., Lee S.D. (2018). Deoxynivalenol- and zearalenone-contaminated feeds alter gene expression profiles in the livers of piglets. Asian-Australas. J. Anim. Sci..

[203] Patil R.D., Sharma R., Asrani R.K. (2014). Mycotoxicosis and its control in poultry: A review. J. Poult. Sci. Technol..

[204] Filazi A., Yurdakok-Dikmen B., Kuzukiran O., Sireli U.T., Manafi M. (2017). Mycotoxins in Poultry. Poultry Science.

[205] Swamy H., Smith T., Cotter P., Boermans H., Sefton A. (2002). Effects of feeding blends of grains naturally contaminated with Fusarium mycotoxins on production and metabolism in broilers. Poult. Sci..

[206] Swamy H.V.L.N., Smith T.K., Karrow N.A., Boermans H.J. (2004). Effects of feeding blends of grains naturally contaminated with Fusarium mycotoxins on growth and immunological parameters of broiler chickens. Poult. Sci..

[207] Girish C., Smith T., Boermans H., Karrow N. (2008). Effects of Feeding Blends of Grains Naturally Contaminated with Fusarium Mycotoxins on Performance, Hematology, Metabolism, and Immunocompetence of Turkeys. Poult. Sci..

[208] Yunus A.W., Blajet-Kosicka A., Kosicki R., Khan M.Z., Rehman H., Böhm J. (2012). Deoxynivalenol as a contaminant of broiler feed: Intestinal development, absorptive functionality, and metabolism of the mycotoxin. Poult. Sci..

[209] Lee J.T., Jessen K.A., Beltran R., Starkl V., Schatzmayr G., Borutova R., Caldwell D.J. (2012). Mycotoxin-contaminated diets and deactivating compound in laying hens: 1. Effects on performance characteristics and relative organ weight. Poult. Sci..

[210] Antonissen G., Van Immerseel F., Pasmans F., Ducatelle R., Janssens G.P.J., De Baere S., Mountzouris K., Su S., Wong E.A., De Meulenaer B. (2015). Mycotoxins Deoxynivalenol and Fumonisins Alter the Extrinsic Component of Intestinal Barrier in Broiler Chickens. J. Agric. Food Chem..

[211] Antonissen G., Croubels S., Pasmans F., Ducatelle R., Eeckhaut V., Devreese M., Verlinden M., Haesebrouck F., Eeckhout M., De Saeger S. (2015). Fumonisins affect the intestinal microbial homeostasis in broiler chickens, predisposing to necrotic enteritis. Veter.-Res..

[212] Wang Y., Quan H., Li X., Li Q., Haque A., Shi Q., Fu Q., He C. (2021). Contamination with Fumonisin B and Deoxynivalenol Is a Threat to Egg Safety and Contributes to Gizzard Ulcerations of Newborn Chickens. Front. Microbiol..

[213] Chen S.S., Li Y.-H., Lin M.-F. (2017). Chronic Exposure to the *Fusarium* Mycotoxin Deoxynivalenol: Impact on Performance, Immune Organ, and Intestinal Integrity of Slow-Growing Chickens. Toxins.

[214] Metayer J.-P., Travel A., Mika A., Bailly J.-D., Cleva D., Boissieu C., Le Guennec J., Froment P., Albaric O., Labrut S. (2019). Lack of Toxic Interaction between Fusariotoxins in Broiler Chickens Fed throughout Their Life at the Highest Level Tolerated in the European Union. Toxins.

[215] Travel A., Metayer J.-P., Mika A., Bailly J.-D., Cleva D., Boissieu C., Le Guennec J., Albaric O., Labrut S., Lepivert G. (2019). Toxicity of Fumonisins, Deoxynivalenol, and Zearalenone Alone and in Combination in Turkeys Fed with the Maximum European Union–Tolerated Level. Avian Dis..

[216] Kolawole O., Graham A., Donaldson C., Owens B., Abia W.A., Meneely J., Alcorn M.J., Connolly L., Elliott C.T. (2020). Low Doses of Mycotoxin Mixtures below EU Regulatory Limits Can Negatively Affect the Performance of Broiler Chickens: A Longitudinal Study. Toxins.

[217] Dwivedi P., Burns R.B. (1984). Pathology of ochratoxin A in young broiler chicks. Res. Vet. Sci..

[218] Dwivedi P., Burns R., Maxwell M. (1984). Ultrastructural study of the liver and kidney in ochratoxicosis A in young broiler chicks. Res. Veter.-Sci..

[219] Dwivedi P., Burns R. (1984). Effect of ochratoxin A on immunoglobulins in broiler chicks. Res. Veter.-Sci..

[220] Dwivedi P., Burns R. (1985). Immunosuppressive effects of Ochratoxin a in young Turkeys. Avian Pathol..

[221] Tamilmani T., Biswas A., Mandal A. (2020). Performance, Immune Response and Blood Biochemical Traits of Broiler Chickens Fed Graded Levels of Dietary Aflatoxin and Ochratoxin Combination. Indian J. Anim. Res..

[222] Zhai S., Zhu Y., Feng P., Li M., Wang W., Yang L., Yang Y. (2021). Ochratoxin A: Its impact on poultry gut health and microbiota, an overview. Poult. Sci..

[223] Weibking T.S., Ledoux D.R., Bermudez A.J., Turk J.R., Rottinghaus G.E., Wang E., Merrill J.A.H. (1993). Effects of Feeding Fusarium moniliforme Culture Material, Containing Known Levels of Fumonisin B1, on the Young Broiler Chick. Poult. Sci..

[224] Weibking T., LeDoux D.R., Bermudez A.J., Turk J.R., Rottinghaus G.E. (1995). Effects on Turkey Poults of Feeding Fusarium moniliforme M-1325 Culture Material Grown under Different Environmental Conditions. Avian Dis..

[225] Bermudez A.J., LeDoux D.R., Rottinghaus G.E. (1995). Effects of Fusarium moniliforme Culture Material Containing Known Levels of Fumonisin B 1 in Ducklings. Avian Dis..

[226] Danicke S., Ueberschar K., Halle I., Matthes S., Valenta H., Flachowsky G. (2002). Effect of addition of a detoxifying agent to laying hen diets containing uncontaminated or Fusarium toxin-contaminated maize on performance of hens and on carryover of zearalenone. Poult. Sci..

[227] Wang E., Norred W.P., Bacon C.W., Riley R.T., Merrill A.H. (1991). Inhibition of sphingolipid biosynthesis by fumonisins. Implications for diseases associated with *Fusarium moniliforme*. J. Biol. Chem..

[228] Murugesan G.R., Ledoux D.R., Naehrer K., Berthiller F., Applegate T.J., Grenier B., Phillips T.D., Schatzmayr G. (2015). Prevalence and effects of mycotoxins on poultry health and performance, and recent development in mycotoxin counteracting strategies. Poult. Sci..

[229] Lumsangkul C., Chiang H.-I., Lo N.-W., Fan Y.-K., Ju J.-C. (2019). Developmental Toxicity of Mycotoxin Fumonisin B1 in Animal Embryogenesis: An Overview. Toxins.

[230] Alaboudi A.R., Osaili T.M., Otoum G. (2022). Quantification of mycotoxin residues in domestic and imported chicken muscle, liver and kidney in Jordan. Food Control.

[231] Antonissen G., De Baere S., Devreese M., Van Immerseel F., Martel A., Croubels S. (2017). Feed contamination with Fusarium mycotoxins induces a corticosterone stress response in broiler chickens. Poult. Sci..

[232] Boermans H.J., Leung M. (2007). Mycotoxins and the pet food industry: Toxicological evidence and risk assessment. Int. J. Food Microbiol..

[233] Tegzes J.H., Oakley B.B., Brennan G. (2019). Comparison of mycotoxin concentrations in grain versus grain-free dry and wet commercial dog foods. Toxicol. Commun..

[234] Thompson A. (2008). Ingredients: Where Pet Food Starts. Top. Companion Anim. Med..

[235] El-Tawaab A.A.A., El-Hofy F.I., Mahmoud A.H., Rashed D.M. (2019). Mycotoxin residues in different chicken products by HPLC and their inactivation using Gamma radiation. Int. J. Pharm. Res. Allied Sci..

[236] Castaldo L., Graziani G., Gaspari A., Izzo L., Tolosa J., Rodríguez-Carrasco Y., Ritieni A. (2019). Target Analysis and Retrospective Screening of Multiple Mycotoxins in Pet Food Using UHPLC-Q-Orbitrap HRMS. Toxins.

[237] Shao M., Li L., Gu Z., Yao M., Xu D., Fan W., Yan L., Song S. (2018). Mycotoxins in commercial dry pet food in China. Food Addit. Contam. Part B.

[238] Leung M.C.K., Díaz-Llano G., Smith T.K. (2006). Mycotoxins in Pet Food: A Review on Worldwide Prevalence and Preventative Strategies. J. Agric. Food Chem..

[239] Rumbeiha W., Morrison J. (2011). A Review of Class I and Class II Pet Food Recalls Involving Chemical Contaminants from 1996 to 2008. J. Med. Toxicol..

[240] Wouters A.T.B., Casagrande R.A., Wouters F., Watanabe T.T.N., Boabaid F.M., Cruz C.E.F., Driemeier D. (2013). An outbreak of aflatoxin poisoning in dogs associated with aflatoxin B1–contaminated maize products. J. Veter.-Diagn. Investig..

[241] FDA (2022). Recalls & Withdrawals. The U.S. Food and Drug Administration. https://www.fda.gov/animal-veterinary/safety-health/recalls-withdrawals.

[242] Glanemann B., Humm K., Pegram C., Chan D.L. (2023). An investigation into an outbreak of pancytopenia in cats in the United Kingdom. J. Veter.-Intern. Med..

[243] Zadravec M., Markov K., Lešić T., Frece J., Petrović D., Pleadin J. (2022). Biocontrol Methods in Avoidance and Downsizing of Mycotoxin Contamination of Food Crops. Process.

[244] Leslie J.F., Moretti A., Mesterházy Á., Ameye M., Audenaert K., Singh P.K., Richard-Forget F., Chulze S.N., Del Ponte E.M., Chala A. (2021). Key Global Actions for Mycotoxin Management in Wheat and Other Small Grains. Toxins.

[245] Nada S., Nikola T., Bozidar U., Ilija D., Andreja R. (2022). Prevention and practical strategies to control mycotoxins in the wheat and maize chain. Food Control.

[246] Dorner J.W. (2009). Biological Control of Aflatoxin Contamination in Corn Using a Nontoxigenic Strain of Aspergillus flavus. J. Food Prot..

[247] Pitt J., Manthong C., Siriacha P., Chotechaunmanirat S., Markwell P. (2015). Studies on the biocontrol of aflatoxin in maize in Thailand. Biocontrol Sci. Technol..

[248] Karunakaran C., Muir W., Jayas D., White N., Abramson D. (2001). Safe storage time of high moisture wheat. J. Stored Prod. Res..

[249] Jayas D.S., White N.D. (2003). Storage and drying of grain in Canada: Low cost approaches. Food Control.

[250] Akhila P.P., Sunooj K.V., Navaf M., Aaliya B., Sudheesh C., Sasidharan A., Sabu S., Mir S.A., George J., Khaneghah A.M. (2022). Application of innovative packaging technologies to manage fungi and mycotoxin contamination in agricultural products: Current status, challenges, and perspectives. Toxicon.

[251] FAO (2004). Food and Agricultural Organization-Worldwide Regulations for Mycotoxins in Food and Feed in 2003. FAO Food and Nutrition Paper No. 81. Rome, Italy. https://www.fao.org/3/y5499e/y5499e0j.htm#bm19.1.8.

[252] CAC. The Codex Alimentarius Commission. General Standard for Contaminants and Toxins in Food and Feed, amended in 2019.

[253] EC (2006). European Commission 2006. Commission Regulation (EC) No 1881/2006 of 19 December as amended, on Setting Maximum Levels of Certain Contaminants in Foodstuffs. https://leap.unep.org/countries/eu/national-legislation/commission-regulation-ec-no-18812006-setting-maximum-levels.

[254] ANVISA (Agência Nacional de Vigilância Sanitária) (2011). Dispõe Sobre Limites Máximos Tolerados (LMT) Para Micotoxinas em Alimentos.

[255] USDA-FAS (2018). USDA Foreign Agricultural Service, GAIN Report Number:CH18026. China Releases Standard for Maximum Levels of Mycotoxins in Foods. https://www.fas.usda.gov/data/china-china-releases-standard-maximum-levels-mycotoxins-foods.

[256] FASA India, Food Safety and Standards Authority of India (2020). First Amendment Regulation Related to Limit of Metal Contaminant, Aflatoxin and Mycotoxin. https://www.fssai.gov.in/upload/notifications/2020/08/5f3d09f97b78aGazette_Notification_Limit_Metal_19_08_2020.pdf.

[257] Li X., Zhao L., Fan Y., Jia Y., Sun L., Ma S., Ji C., Ma Q., Zhang J. (2014). Occurrence of mycotoxins in feed ingredients and complete feeds obtained from the Beijing region of China. J. Anim. Sci. Biotechnol..

[258] South African Government (2020). Farm Feeds Regulations: Amendment. Department of Agriculture, Forestry and Fisheries No.R.70. https://www.gov.za/sites/default/files/gcis_document/201409/3293570.pdf.

